# TRANSIT - A Software Tool for Himar1 TnSeq Analysis

**DOI:** 10.1371/journal.pcbi.1004401

**Published:** 2015-10-08

**Authors:** Michael A. DeJesus, Chaitra Ambadipudi, Richard Baker, Christopher Sassetti, Thomas R. Ioerger

**Affiliations:** 1 Department of Computer Science, Texas A&M University, College Station, Texas, United States of America; 2 Department of Microbiology and Physiological Systems, University of Massachusetts Medical School, Worcester, Massachusetts, United States of America; University of Canterbury, NEW ZEALAND

## Abstract

TnSeq has become a popular technique for determining the essentiality of genomic regions in bacterial organisms. Several methods have been developed to analyze the wealth of data that has been obtained through TnSeq experiments. We developed a tool for analyzing Himar1 TnSeq data called TRANSIT. TRANSIT provides a graphical interface to three different statistical methods for analyzing TnSeq data. These methods cover a variety of approaches capable of identifying essential genes in individual datasets as well as comparative analysis between conditions. We demonstrate the utility of this software by analyzing TnSeq datasets of *M. tuberculosis* grown on glycerol and cholesterol. We show that TRANSIT can be used to discover genes which have been previously implicated for growth on these carbon sources. TRANSIT is written in Python, and thus can be run on Windows, OSX and Linux platforms. The source code is distributed under the GNU GPL v3 license and can be obtained from the following GitHub repository: https://github.com/mad-lab/transit

This is a *PLOS Computational Biology* Software paper

## Introduction

**Fig 1:**
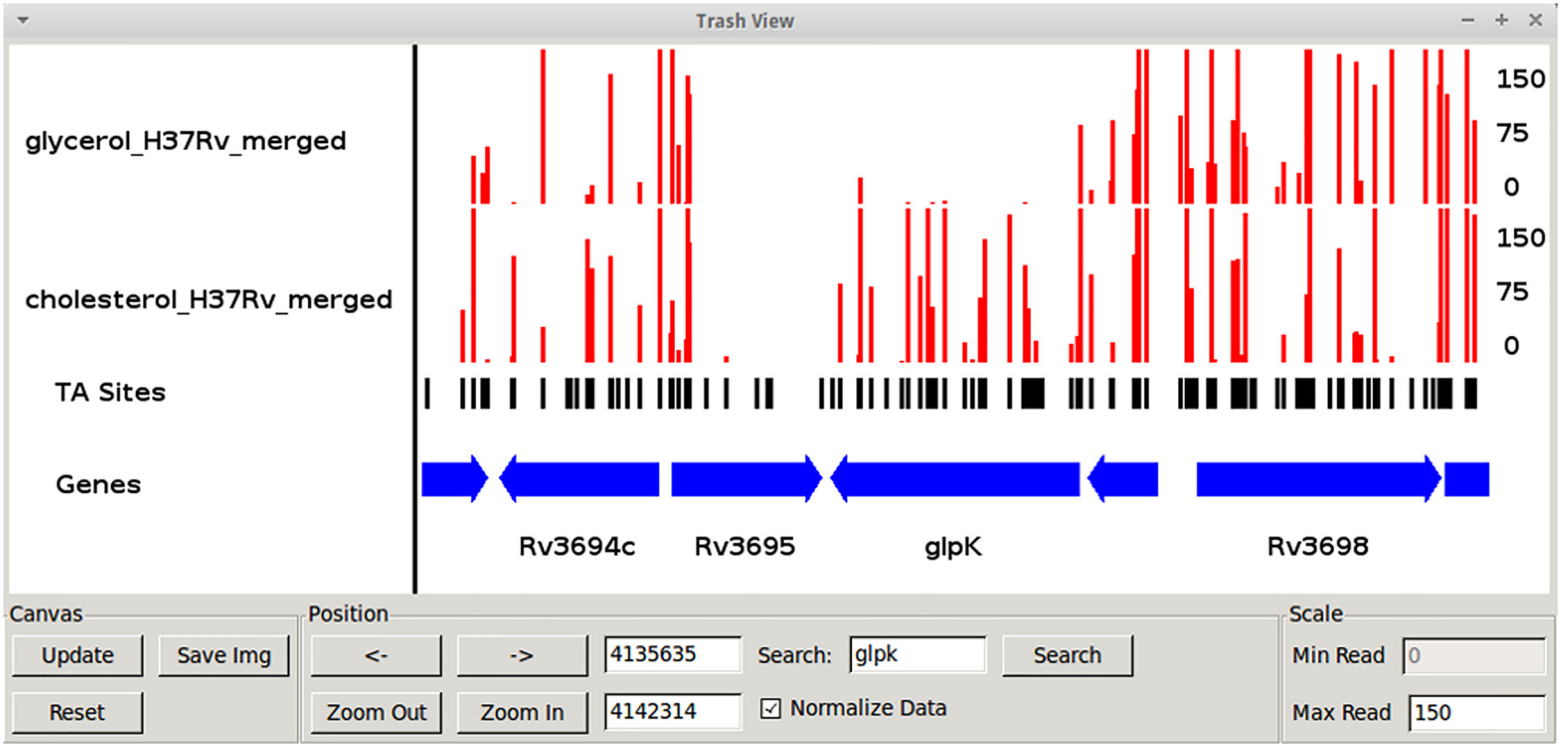
Track View of read counts for datasets grown in glycerol and cholesterol. This region spans approximately 12 kb, and includes 5 genes. TA dinucleotides, which are candidate insertion sites, are indicated in the middle track. Vertical height of each bar reflects # of reads or Tn insertions at each TA site. Some sites with no insertions are probably missing from the library, while others may reflect essential regions. Note that GlpK lacks insertions in the glycerol condition, indicating that it is essential when grown on glycerol.

Transposon insertion sequencing (TnSeq for short) is a popular experimental methodology for determining essential (and conditionally essential) regions in bacterial genomes [1]. TnSeq (in the broad sense used in this paper) refers to a family of related methods that use deep sequencing to survey a transposon insertion library and quantify the abundance of insertions at different sites in the genome [2–5]. The specific methodologies differ in the details of library preparation (such as use of shearing versus digestion, method of enrichment, or the choice of transposable element) [6]. While there are several tranposons that can be used to construct Tn insertion libraries, one of the most commonly used is the Himar1 transposon, which is used in several specific protocols including HITS [3], Tn-seq [4], and INSeq [2]. The Himar1 transposon inserts at random TA dinucleotide sites during the library generation process [7]. Depending on size of gene and GC-content there are typically between 5 to 50 TA sites per gene. Essential regions are inferred by the lack of insertions observed in a region (presumably because the insertion of the transposon (Tn) disrupts the protein product, making it non functional). Conditionally essential regions have insertions in one condition but not in another (See Fig 1). Knowledge of (conditionally) essential genes plays an important role in drug discovery, as these could be drug targets, and the ability to detect conditionally essential genes is helpful in working out pathways (e.g. comparative analysis between samples with and without supplementation of a critical metabolite).

The preparation of TnSeq samples for sequencing involves fragmenting genomic DNA, attaching sequencing adapters, and amplifying with PCR primers to enrich the sample for fragments carrying Tn:genomic junctions. Illumina next-generation sequencers are the most frequently used platform to sequence TnSeq libraries. The datasets generated from an Illumina sequencer contain short reads (∼ 100 bp) that have the terminus of the Tn as a prefix and a genomic suffix that can be mapped (aligned) to the genome to identify which TA site they represent.

While the relative abundance of insertion mutants can be estimated based on the frequency of read counts or template counts corresponding to an insertion site, stochastic effects during amplification and library generation can also influence these measurements. Despite these fluctuations, some regions show systematically suppressed (or inflated) counts, which could reflect a gene whose disruption causes a growth defect (or growth advantage). In addition, there can also be missing sites not represented in the library. Analysis of TnSeq data is challenging, especially with low density libraries where there are large number of TA sites not represented in the library. Several methods have been proposed for rigorously quantifying the statistical significance of essential regions, including models using the Negative Binomial distribution [8] and the Poisson distribution [9], a non-parametric test based on re-sampling counts within a sliding window [10], Bayesian methods [11] and Hidden Markov Models [12, 13].

TRANSIT is a new software tool that automates the analysis of Himar1 TnSeq datasets. It has a graphical interface that allows a user to load TnSeq datasets and apply several built-in analyses to identify essential (and conditionally essential) regions, calculate statistical significance, and visualize the results in different ways. For essentiality analysis, TRANSIT provides two alternative methods: a Bayesian method that quantifies the significance of “gaps” (or long consecutive sequences of TA sites lacking insertions) [11], complemented by a Hidden Markov Model (HMM) that also incorporates local differences in read counts [13]. For comparative analysis, TRANSIT utilizes a permutation test that compares the difference of the counts in a genomic region between two different conditions to determine if there is a statistically significant difference (e.g. putatively reflecting selection for or against disruption in one of the two conditions). A pre-processor called TPP (for TRANSIT Pre-Processor) is provided which extracts read counts from raw sequence data files (in .fasta, .fastq or fastq.gz format), maps them to the reference genome, optionally reduces them to unique template counts, and outputs them in .wig format for loading into TRANSIT. Finally, numerous statistics are generated for analyzing the quality of TnSeq datasets and diagnosing any potential problems (i.e. with the library or sample preparation).

TRANSIT was initially designed to analyze TnSeq libraries prepared by the protocol in [14], which uses a custom barcoding scheme (unique nucleotides which occur in read 2). TPP has a default mode to recognize these barcodes and use them to reduce read counts to template counts, as described below. However, TRANSIT was intentionally designed in a modular way to decouple the preprocessing from the computational analysis to allow the statistical analysis tools of TRANSIT to be applied to datasets obtained from other (Himar1) TnSeq protocols [2–4]. For example, if a dataset is collected with just single-ended reads, or a protocol without barcoding was used for sample preparation, TPP can configured to simply process read 1 without read 2. If an alternative barcoding scheme were used, TPP might have to be modified, or users can implement their own processing pipeline for mapping reads and quantifying insertions at genomic locations. As long as these counts are written out as intermediate files in .wig format, they can be input to TRANSIT for subsequent statistical analysis. TRANSIT could in principle be modified to analyze other TnSeq libraries such as those generated with the Tn5 transposon [5].

Several other software tools have been developed for analysis of TnSeq data. Some are purely computational [12, 15] and do not have the convenient graphical features of TRANSIT, such as TrackView (to display insertion patterns at various loci) or Volcano plots (to visualize the distribution of hits in comparative analysis). The most similar alternatives are ESSENTIALS [8] and Tn-seq Explorer [16]. ESSENTIALS uses the Negative Binomial distribution to identify essential genes and quantify their statistical significance. However, it has been observed to output an excessive number of essential genes when utilizing its reported p-values for classification [16] and can be susceptible to insertions in the N- or C- terminii, causing essential genes to appear to be non-essential [11]. Tn-seq Explorer uses a sliding window approach to identify regions where there is a deficit of reads relative to the rest of the genome. However, there is no calculation of statistical significance for the Essentiality Index (EI) computed for each gene. The permutation test in TRANSIT also provides a more statistically rigorous way to identify conditionally essential genes (comparative analysis between conditions), in contrast to the simple comparison of EI values in Tn-seq Explorer.

## Design and Implementation

### Analysis Methods

TRANSIT provides several statistical methods capable of accomplishing two common types of tasks:

1. Identifying essential genes in a single growth condition
  - Bayesian/Gumbel Method
  - Hidden Markov Model
2. Identifying conditionally essential genes between conditions (comparative analysis)
  - Resampling (permutation test)

All TnSeq analysis methods are sensitive to insertion density or library saturation (to different degrees). It is important to have sufficient diversity (or saturation) of the Tn mutant library, so that as many of the TA sites in non-essential regions are represented as possible. While saturation rarely achieves 100%, good libraries often have density greater than 50%, whereas libraries with lower density in the 20-30% range are more challenging to analyze and give less confident predictions (because a sequence of TA sites could be missing insertions due to chance).

#### Bayesian/Gumbel Method

For analyzing essentiality in single conditions, TRANSIT incorporates a Bayesian method that identifies the longest consecutive sequence of TA sites lacking insertion in a gene (or “gap”), and calculates the probability of this using the Gumbel or Extreme Value distribution [11]. The basis of this approach is that, in non-essential regions, gaps will occur by chance (depending on degree of saturation), and the probability of a long gap decreases geometrically. Thus essential genes can be recognized by unusually long gaps, and the posterior probability of the longest gap can be calculated using a Bayesian formula. The Bayesian formula is a joint probability density function Eq (1) with unobservable parameters that must be estimated using the Metropolis-Hasting sampling algorithm [11]. In the end, the Bayesian method calculates a posterior probability of essentiality (called ${\bar{Z}}_{i}$) for each gene. While p-values are not traditionally used in a Bayesian framework, a technique for controlling the false-discovery rate is used to select a threshold of posterior probabilities that can be used to determine a set of confident essentials and non-essentials that is adjusted for multiple comparisons [17]. Some genes might be labeled as Uncertain, which is important if data is too sparse or the gene is too short to make a confident call. While the Gumbel method does not take into consideration the magnitudes of the read counts, an important advantage of this analysis is that it is not sensitive to a few insertions at the N- and C-termini of essential genes (since there is often still a large gap in the middle of an essential ORF), or insertions in non-essential linker regions between domains, and it can identify “domain-essentials”, which are genes containing both an essential and non-essential domain.

$$\begin{array}{ccc} & & p\left(Y,Z,\phi_{0},\omega_{1}\right)=p\left(Y|Z,\phi_{0},\omega_{1}\right)\times \pi \left(\phi_{0}\right)\times \pi \left(Z|\omega_{1}\right)\times \pi \left(\omega_{1}\right)\\ & & \;=[\prod_{i=1}^{non}Gumbel\left(r_{i}|\mu ,\sigma \right)\times N\left(s_{i}-\lambda_{r}r_{i},\sigma_{r}^{2}\right)]\\ & & \;\times [\prod_{i=1}^{ess}\Omega \left(s_{i}\right)\times N\left(r_{i}-\lambda_{s}s_{i},\sigma_{s}^{2}\right)]\times Beta\left(\phi_{0};\alpha_{0},\beta_{0}\right)\\ & & \;\times Bin\left(K_{z};G,\omega_{1}\right)\times Beta\left(\omega_{1};\alpha_{w},\beta_{w}\right) \end{array}$$ (1)

In this formula (from [11]), *Y*_*i*_ represents the observed insertions patterns in each gene, *Z*_*i*_ is a binary variable that indicates whether each gene is essential, and the other variables are internal parameters of the model that get estimated through the sampling process.

#### Hidden Markov Model

To complement the Bayesian method, TRANSIT also offers an HMM to identify essential regions in a *non-gene-centric* way (not limited by ORF boundaries) [13]. Thus the HMM can be used to identify essential loci larger than one gene, e.g. an operon, or smaller, e.g. an essential protein domain. Also it can potentially identify essential intergenic regions [10].

**Fig 2:**
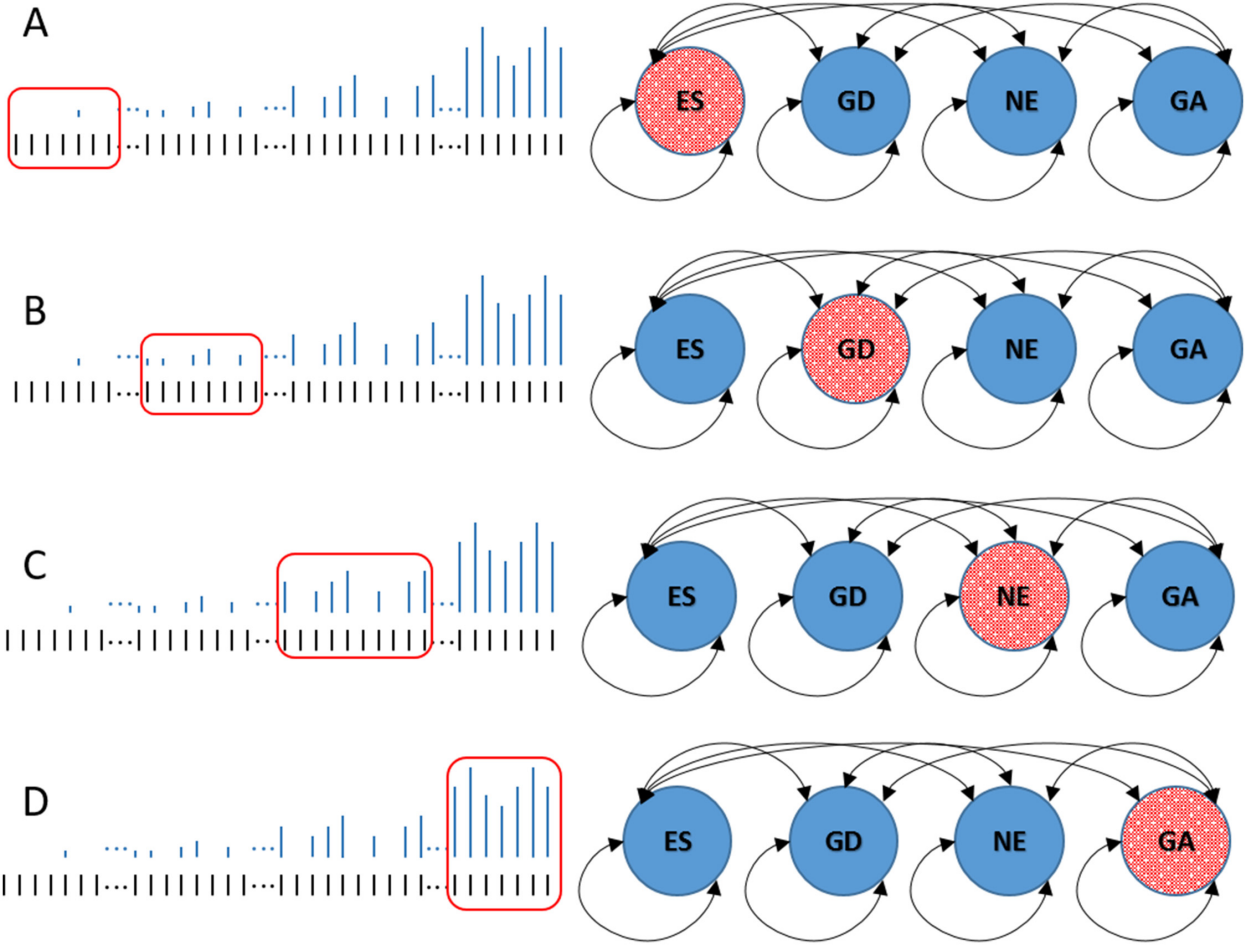
Hidden Markov Model Diagram. The HMM is fully connected, allowing transitions between each of the states. Transition probabilities and parameters are estimated in such a way that the HMM will remain in the state which best represents the read-counts observed. (a) Essential regions (“ES”) are mostly devoid of insertions, (c) while non-essential regions (“NE”) contain read-counts around the global mean. (b) Growth-defect regions (“GD”), and (d) growth-advantage regions (“GA”) represent those areas with significantly suppressed or inflated read-counts.

An HMM is a popular choice for analyzing sequential data. In this context, the HMM is applied to the sequence of TA sites to obtain the most probable state (essentiality) assignment based on the read count at the site and the distribution over the surrounding sites. In this manner, the HMM enforces a local consistency among the state assignments, despite not explicitly using a sliding window. The HMM in TRANSIT is a 4-state model (See Fig 2) that with states for: a) essential regions (ES), b) non-essential regions (NE), c) growth-defect regions (GD, with suppressed read counts), and d) growth advantaged regions (GA; with excess read counts, inflated above the global mean). The likelihood of read counts in each state is determined by a geometric distribution (based on the observation that low read counts are more frequent and sites with higher counts are more rare), where the mean is near-0 for ES, near the global mean for NE, intermediate for GD, and high for GA.

The parameters of the HMM (transition probabilities, etc.) are dynamically adjusted to the attributes of the dataset (such as insertion density and mean read count) in such a way that the model tends to remain within a state (despite a few sites that may not fit) until enough evidence accumulates to justify a transition to another state (See Fig 2). Given the transition probabilities and other parameters of the model, the state distributions for each TA site are estimated from the observed counts using the Viterbi algorithm [18]. The HMM has been shown to perform well and make reasonable essentiality calls even in datasets with density as low as 20% [13]. At the end of the analysis, the proportions of sites labeled by each of the 4 states is reported. Typically around 15% of the genome would be expected to be essential in most bacteria [19], and most of the rest of the genome would be non-essential, while only a small fraction (on the order of 5-10%) might be labeled as GD or GA. An example of a putative GA region would be one containing virulence genes, which are required *in vivo* for infection but are often lost *in vitro* because of the taxing energy requirements on the organism. As a post-processing step, the essentiality state of each gene is called based on the labeling of the majority of TA sites within the ORF.

#### Resampling

For comparative analysis, TRANSIT uses a variation of the classical permutation test in statistics [20]. For each gene, the read counts at all the TA sites and all replicates in each condition are summed, treating replicates within a condition as independent and identically distributed. The difference between the sum of read-counts at each condition is then calculated. The significance of this difference is evaluated by comparing to a resampling distribution generated from randomly reshuffling the observed counts at TA sites in the region among all the datasets. This creates a distribution of read count differences that might be observed by chance, assuming a null hypothesis that the two conditions are not in fact different. A p-value is then derived from the proportion of reshuffled samples that have a difference more extreme than that observed in the actual experimental data.

**Fig 3:**
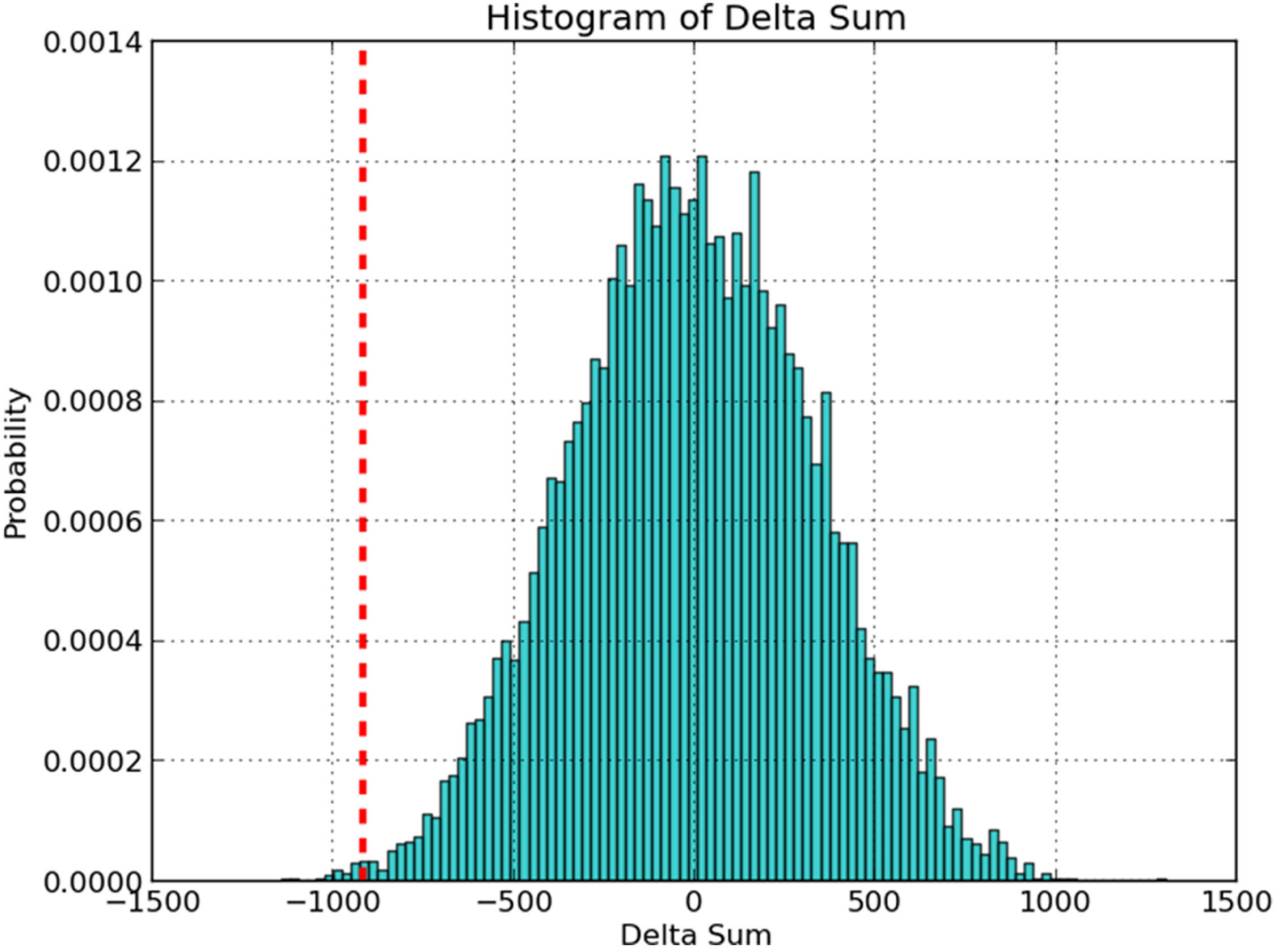
Resampling histogram for gene Rv0017c. Rv0017c has 23 TA sites, and the sum of the observed counts at the TA sites in this genes *in vitro* was 1,318 and *in vivo* was 399, therefore the observed difference in counts is -918. To determine the significance of this difference, 10,000 permutations of the counts at the TA sites among the datasets was generated and the observed differences plotted as a histogram showing that a difference as extreme as -918 almost never occurs by chance. The p-value is determined by the tail of this distribution to be 0.003 (30 out of 10,000).

Due to the stochastic nature of read counts, there will almost always be some measurable difference between these sums. If this difference in sums of read counts falls within the bounds of the resampling distribution, this is interpreted as being due to chance. On the other hand, true conditionally essential genes will show a highly significant difference as insertions in the locus will be observed in one condition but not the other, resulting in a difference which is typically much larger than any of the differences observed by randomly re-shuffling. Furthermore, this method can detect genes whose disruption leads to a reduction in fitness; that is, genes which are not absolutely essential in one of the conditions, but instead have lower read-counts in one of the conditions compared to the other. The permutation test distinguishes which of these differences is statistically significant. p-values are derived from the fraction of samples that exceed the observed difference (See Fig 3), and this is adjusted for multiple comparisons by the Benjamini-Hochberg procedure.

The permutation test requires that the datasets be comparably normalized. TRANSIT provides several alternative ways to normalize TnSeq data, each with different strengths and weaknesses in dealing with various sources of noise in real datasets. The default normalization procedure is the non-zero mean (NZmean) method to normalize datasets to have the same mean over non-zero sites. In our experience, this is better than normalizing by the total read-counts, which is sensitive to the degree of saturation of a library. The normalization is achieved by dividing by the total number of reads in the dataset by the total number of sites with at least one insertion, and using this (and the desired mean) as a scaling factor:

$$\begin{array}{c} \sigma_{j}=\mu_{g}\times \frac{\text{Number}\;\text{of}\;\text{sites}\;\text{with}\;\text{an}\;\text{insertion}}{\text{Total}\;\text{number}\;\text{of}\;\text{reads}\;\text{in}\;\text{dataset}\;j} \end{array}$$

where *μ*_*g*_ is the global mean read-count across all datasets. The normalized counts at each site *i* in dataset *j* are the raw counts times the scaling factor ($c_{ij}^{\prime }=c_{ij}\times \sigma_{j}$).

### Pre-Processing

TRANSIT takes .wig files as input, which contain counts of reads (or unique templates) observed at each each TA site. In this way, TRANSIT accepts datasets prepared with any pre-processing or custom protocol. An optional pre-processor called called TPP is provided with the software distribution for extracting these counts from raw sequencing files (typically in .fastq format, paired-end reads in two files called “read1” and “read2”). Note that this pre-processing procedure is designed for libraries prepared in adherence with the protocol described in [14]. However, other labs might want to apply their own custom pre-processing procedure, particularly if they use an alternative protocol for preparing TnSeq samples for sequencing or if they use a different transposon.

**Fig 4:**
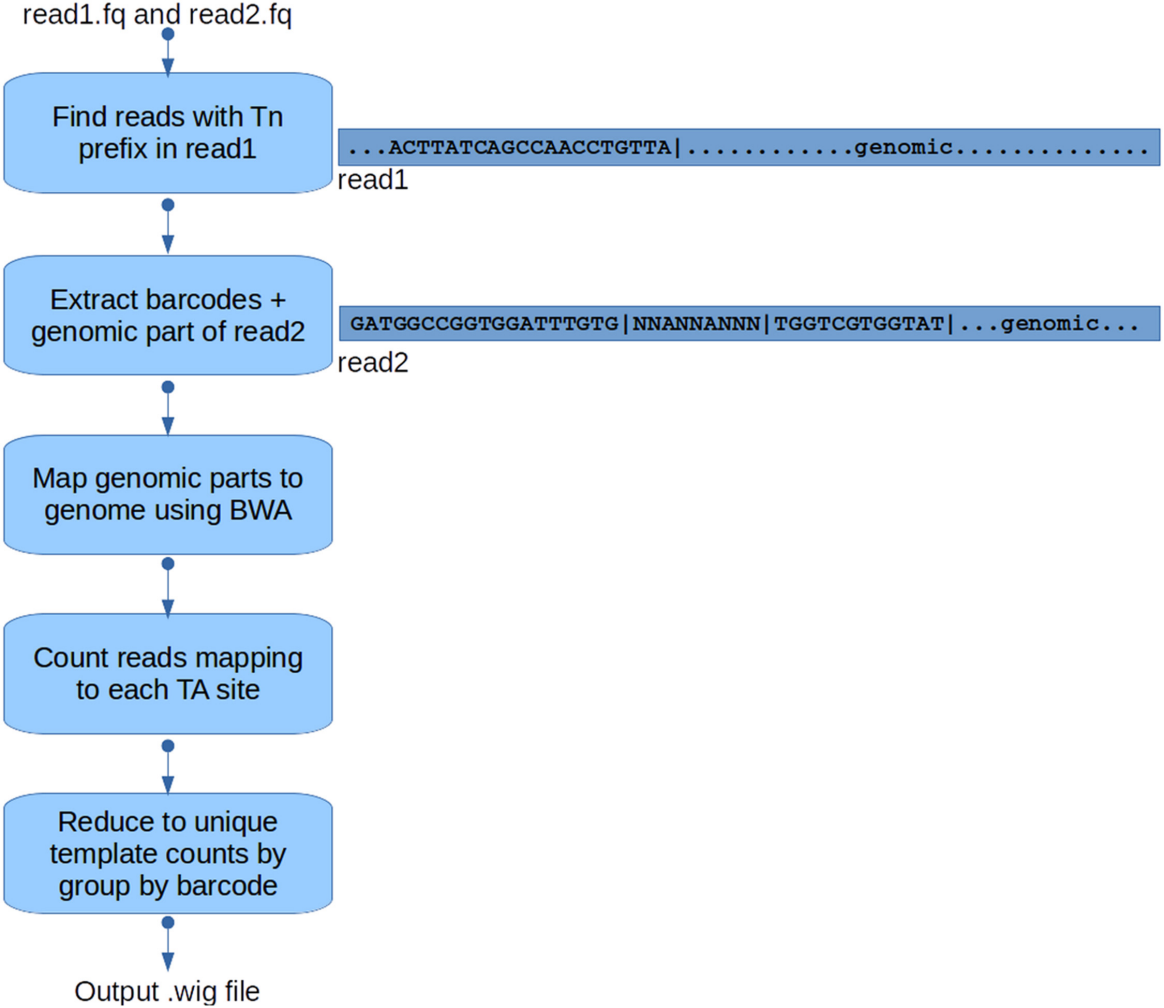
TPP flowchart. Reads in .fasta, .fastq or fastq.gz format are taken in as input, and mapped to the genome to get read-counts at individual TA sites. A .wig formatted file is returned as output, containing the coordinates and the read-counts at all TA sites in the genome.

TPP uses BWA (Burroughs-Wheeler Aligner; [21]) to map reads into the genome. The workflow performed by TPP (Fig 4) can be briefly summarized as follows: First, read 1 is analyzed to identify the subset of reads that have a prefix matching the terminus of the Himar1 transposon (ACTTATCAGCCAACCTGTTA). The transposon prefix is stripped off, and the genomic suffix is mapped onto the genome to identify the TA site (and strand) represented by each read. Then, read 2 is analyzed to extract both a random nucleic-acid barcode (See Fig 4) and genomic suffix. The genomic suffix is also mapped onto the genome and represents the end-point of the original DNA fragment (typically a few hundred bp away). All the reads mapping to a given TA site are reduced to unique “template” counts by discarding duplicates that have the same barcode and end-point, and these template counts are written out in .wig format (for input to TRANSIT). (If barcodes were not applied during sample prep or only single-ended data was collected, TPP can be run optionally without providing read 2, in which case raw read counts are output, without reduction to unique template counts.) In our experience, this barcoding data-reduction technique has proved useful in reducing PCR effects, which can artificially bias the read counts depending on which fragments amplify more efficiently. We have observed that this is especially true for noisier datasets, where read counts are highly variable (though recent optimizations in the PCR protocol have mitigated this problem somewhat [10]) and the resulting template counts better reflect the true abundance of distinct mutants in the population. It can take on the order of an hour to process each dataset (depending on size of dataset as well as speed of computer), which is dominated by the time required to align the genomic parts of the reads to the genome using BWA. Ideally, it is recommended to collect datasets with 5-10 million pairs of reads, which often gets reduced to ∼ 2 million unique templates, in order to have sufficient dynamic range of counts for analysis (for example, aiming for a nominal mean of ∼ 50 templates per TA sites, estimated based on ∼ 75,000 TA sites in the *M. tuberculosis* genome, with library saturation of around 50%).

Multiple statistics are calculated by TPP for diagnostic purposes. The primary metrics used to assess the quality of a TnSeq dataset are insertion density and mean read count, which should be *P*_*ins*_ > 30% and NZmean > 10. Additional statistics are reported, such as number of reads with valid Tn prefixes, number of reads mapping to genome (broken down into read 1, read 2, and both), correlation of reads at each TA sites on forward versus reverse strand, ratio of reads to templates, etc. These metrics are important for diagnostic purposes. In addition, specific nucleotide sequences representing the vector or primer are counted. If the number of mapped reads is low, then the user could check to see if there is a large fraction of reads matching these sequences, which could indicate phage contamination in the library (left over from the original transfection in constructing the library) or excessive primer-dimers lacking genomic inserts generated during sample preparation.

### Interface

**Fig 5:**
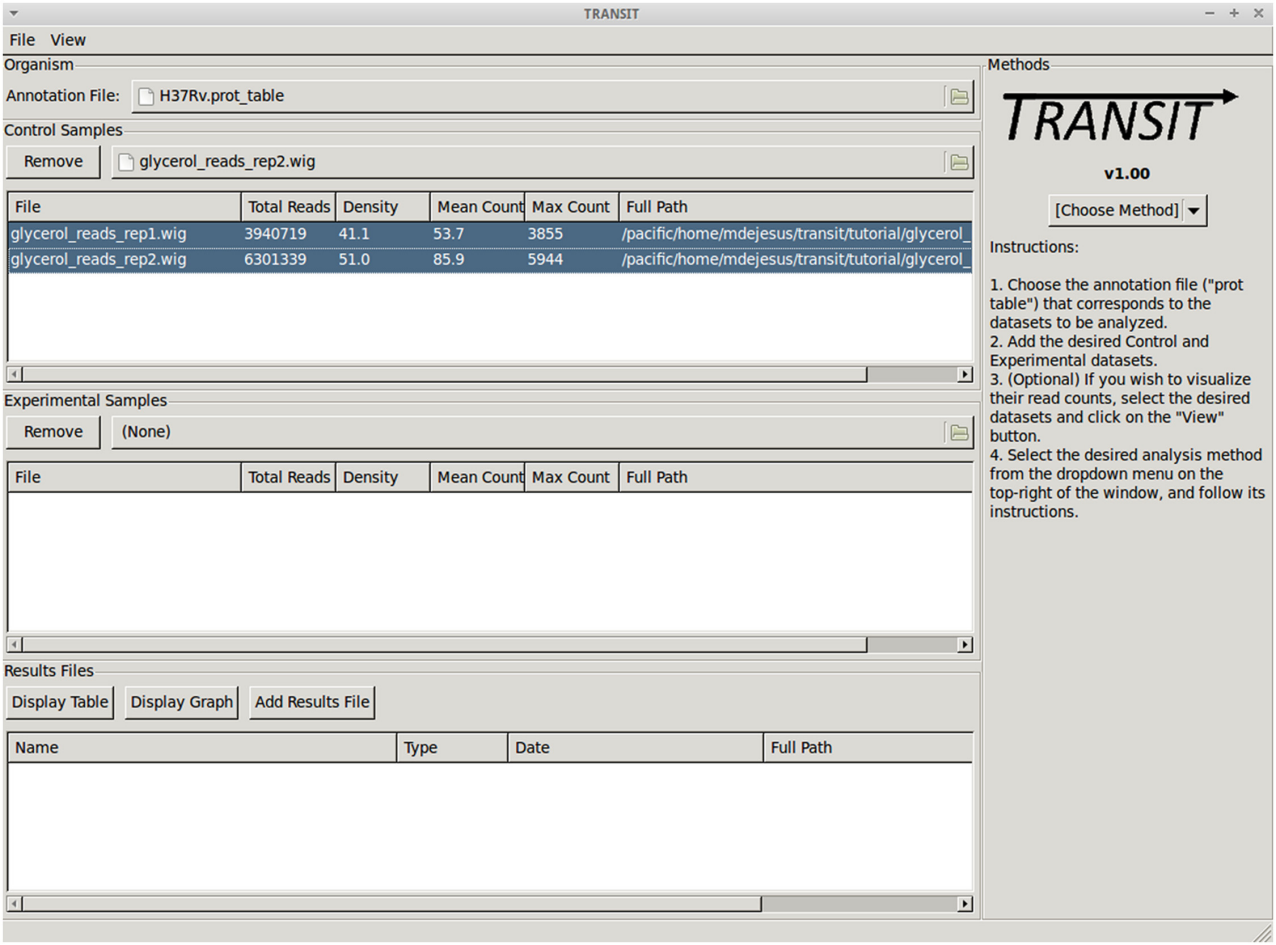
Picture of the main TRANSIT interface.

The main TRANSIT interface (Fig 5) allows the user to select an annotation file (in a tab-separated format called “.prot_table”), which contains the definitions of genes and their coordinates. It must match the reference genome to which reads were mapped by TPP. Several .prot_tables are provided online for commonly used genomes, and a script for converting annotations for other organisms from Genbank to .prot_table format is also available.

From the main interface the user can also load datasets in .wig format (e.g. output by TPP), which contain the coordinates and template counts at TA coordinates throughout the genome. Once a dataset is loaded, the corresponding table will be populated with diagnostic information about the dataset, like density and mean read count. This information can be used to compare datasets and identify potential problems. TRANSIT also provides a way to create a scatter plot of the read counts in two datasets from the menu-bar at the top of the interface. In addition, the user can visualize read-counts throughout the genome using TRANSIT’s Track View (also found in the menu-bar; see Fig 1).

Once the user has picked the desired datasets for comparison, they can choose which analysis they wish to perform from the drop-down menu to the right. As soon as a method is selected, the right-hand panel of TRANSIT’s interface is automatically populated with the appropriate parameters for the method selected. The user may use the default parameters (which are intended to work well on most datasets) or change individual parameters as needed. Parameter definitions are provided in the documentation included with TRANSIT.

**Fig 6:**
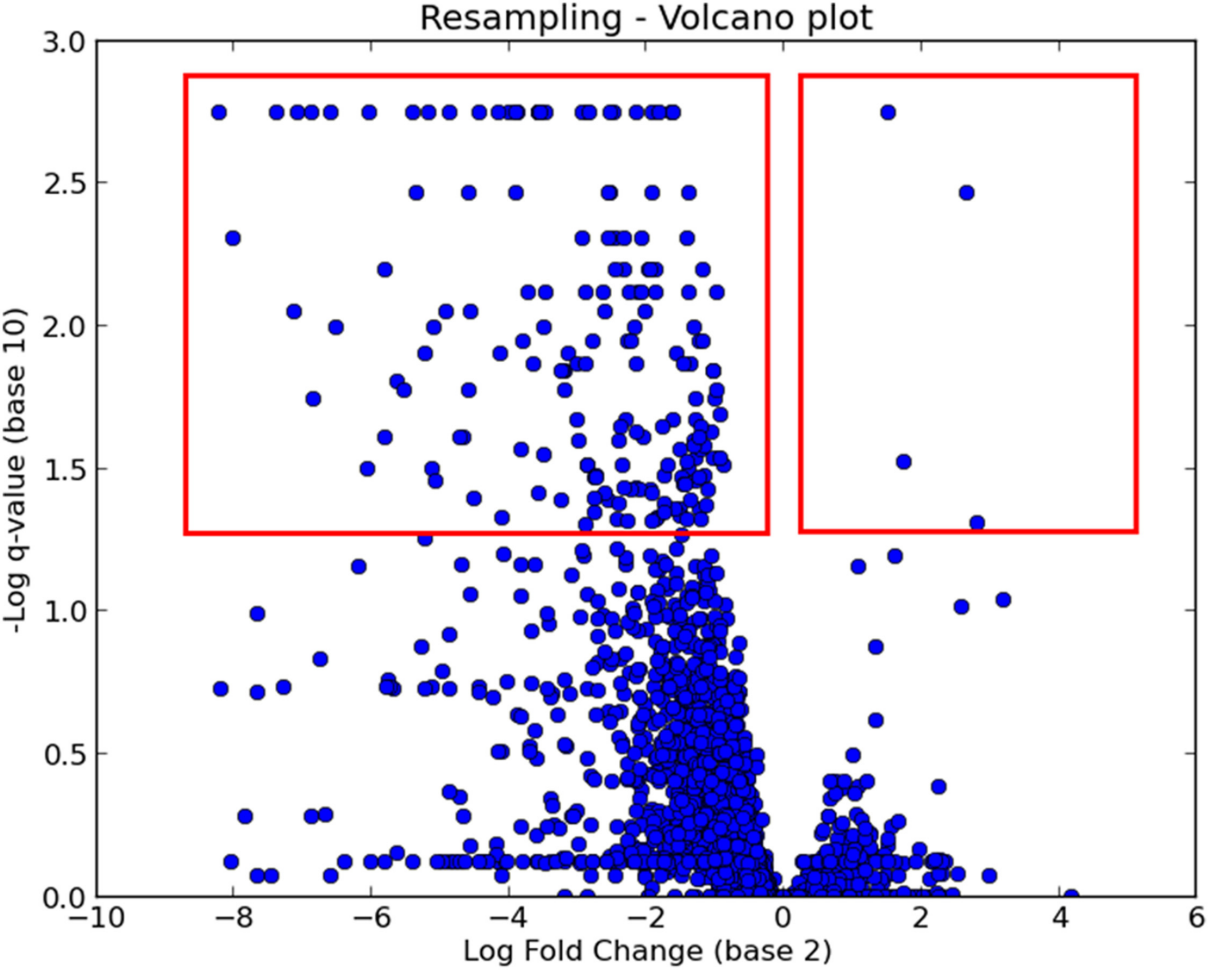
Volcano plot of resampling results comparing replicates grown *in vitro* versus *in vivo*. Significant hits have *q* < 0.05 or −*log*_10_ *q* > 1.3. Note that some genes have increased essentiality (fewer insertions; left side) and some decreased essentiality (right side).

After TRANSIT completes an analysis, it will create output files in the specified location and automatically add them to a list in the results window to keep track of the results files created in a session. Output files are tab-separated so they can be opened in the user’s preferred spreadsheet software (e.g. Excel). TRANSIT also has the capability of opening results files in a new window, by selecting them from the list of results files and clicking on the “Display Table” button. This list also allows the user to generate custom graphs of the results, such as volcano plots (which plots log-fold change in read counts and adjusted p-values; See Fig 6).

**Fig 7:**
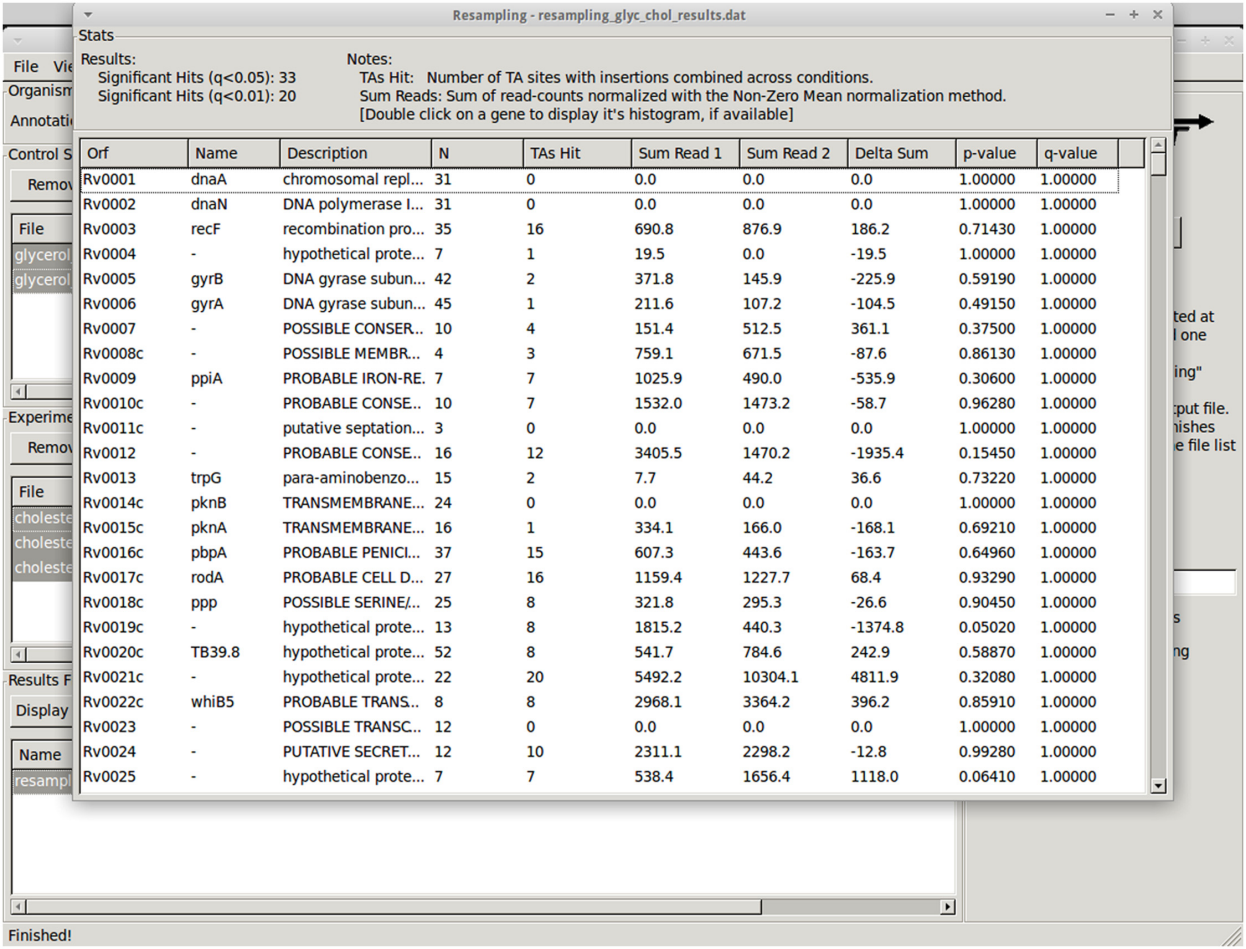
Table of results obtained from resampling, comparing replicates grown in glycerol versus cholesterol.

The results window (Fig 7) allows the user to sort on the desired column (e.g. p-values) thus facilitating the identification of genes of interest. The user can right-click on a gene to display the gene in Track View to examine the insertion patterns (Fig 1), or get other method-specific options (like histograms of the permutations obtained with the resampling method; See Fig 3).

## Results

We illustrate the utility of TRANSIT by analyzing several published TnSeq datasets of *M. tuberculosis* H37Rv as well as *H. influenza*. The H37Rv strain has a total of 74,605 TA sites distributed randomly throughout the genome. It has 3989 genes with an average of 14 TA sites per gene, with almost all genes containing at least one TA site. The TnSeq datasets analyzed came from libraries grown on glycerol, a common *in vitro* carbon source, and cholesterol, a carbon source required for infection [22]. Datasets were obtained in multiple replicates, with two replicates grown on glycerol, and three replicates grown on cholesterol. The insertion density of the replicates was in the range of 40% to 60%, with mean template-counts ranging from 50-90 per TA site.

### Bayesian/Gumbel Method

To identify essential genes we analyzed the datasets grown on glycerol using the Bayesian/Gumbel Method, which performs an analysis on an individual condition. After loading the glycerol replicates and the annotation file into TRANSIT, and running the Gumbel method with default parameters, we obtained an output file with results.

A total of 674 genes was found to be essential (${\bar{Z}}_{i}>\theta_{e}$, where *θ*_*e*_ is a threshold determined by the method used to control the FDR) by the Gumbel method (16.3%), matching expectations that typically 15% of bacterial genomes are necessary for growth [19, 23]. The Gumbel method also identified 2670 non-essential genes (${\bar{Z}}_{i}<\theta_{n}$), with the remainder being labeled as Uncertain (because the posterior probability did not exceed the significance thresholds), or were too short for reliable analysis. Table 1 contains a summary of the classifications obtained by the Gumbel method.

**Table 1:** Table of Bayesian/Gumbel Results for H37Rv grown in glycerol.

Type of Gene	# of Genes
Essential	674
Uncertain	307
Non-Essential	2670
Too Small	338
Total	3989

Breakdown of essentiality calls for the glycerol datasets obtained by the Bayesian/Gumbel method. Essential and Non-Essential genes are those genes whose posterior probability of essentiality exceeds the dynamic thresholds of essentiality. Uncertain genes are those who do not exceed these thresholds, and “Too Small” represents those genes who are too small for reliable analysis.

Well-known essential genes like GyrA (DNA gyrase A) and RpoB (DNA-directed RNA-polymerase) were identified as essential by the Gumbel method, both achieving a posterior probability of essentiality of 1.0. Those genes are completely devoid of insertions (aside from a few insertions at the N/C termini). However, one of the strengths of the Gumbel method is that it can also identify genes which contain both essential and non-essential regions, indicative of essential domains. An example of such a gene is Rv3910, which codes for an essential MviN domain [24]. TRANSIT identifies this gene as essential, as it contains a large gap of 32 TA sites in a row without insertions, despite the fact that it has insertions on 10 out of the the remaining 17 sites. DeJesus et al. [11] discusses concordance of these results with previous essentiality analysis using the hybridization-based TraSH method [25].

### Hidden Markov Model

Another approach to analyzing an individual condition is the Hidden Markov Model. Like before, glycerol replicates were loaded into TRANSIT and the HMM method was run using default parameters.

The HMM analysis classified 16.3% of the sites in the genome as belonging to the “Essential” state, 5.4% belonging to the Growth-Defect state, 77.1% to the Non-Essential state, and 1.2% to the Growth Advantage state (See Table 2). One advantage of this method is that it is not limited to gene-boundaries but instead can assess essentiality of entire regions. For example, the PDIM locus (*fadD26*, *ppsABCDE*, *mas*; which spans ∼ 38kb, 594 TA sites, and 10 genes) is required for virulence *in vivo* but has high metabolic costs for the organism *in vitro* and therefore results in a Growth-Advantage for the organism when disrupted. Indeed, sites in this region (e.g. Rv2930-Rv2939) are labeled “GA” (the mean read count in this region is 502.0, a 1.9 fold increase from the global mean), thus identifying that disruption of the PDIM locus when growing on standard *in vitro* conditions affords an advantage to the organism.

**Table 2:** Table of HMM Results for H37Rv grown in glycerol.

Type of Region	% of TA Sites (out of 74,605)
Essential	16.3%
Growth Defect	5.4%
Non-Essential	77.1%
Growth Advantage	1.2%

Distribution of state calls for the glycerol datasets obtained by the HMM method. Essential states represent those regions which are mostly devoid of insertions. Non-Essential regions contain read-counts that are close to the mean read-count in the dataset. Growth-Defect regions and Growth-Advantage regions represent those regions which have significantly suppressed or increased read-counts.

### Resampling

The resampling method can be used for comparative analysis of different growth conditions. After adding glycerol replicates as Control datasets and cholesterol replicates as Experimental datasets, the resampling method was run with default parameters.

A total of 28 genes were identified as differentially essential (adjusted p-value < 0.05; see Table 3 for the number of conditionally essential genes identified). Several of these genes are known to be uniquely required for growth in glycerol or cholesterol. For example, glycerol kinase (GlpK) is necessary for growth on glycerol but should not be necessary for growth on other carbon sources like cholesterol. Indeed, GlpK had a total of 1968 reads in the cholesterol condition, and only 22 total reads in glycerol, achieving an adjusted p-value (or q-value) of 0.0 with the resampling method.

**Table 3:** Table of results for comparative analysis between glycerol and cholesterol.

Type	Count
# of genes with *q* < 0.05 in resampling	28
# of genes essential in glycerol but not cholesterol	8
# of genes essential in cholesterol but not glycerol	20

Breakdown of the number of differentially essential genes identified by the resampling method, in each condition (glycerol and cholesterol). Differentially essential genes are those with an adjusted p-value *q* < 0.05.

Among those genes identified as necessary for growth in cholesterol were several of the Mce-family of proteins, which is believed to be involved in lipid catabolism [26]. These included Mce4A, Mce4C, Mce4D, and Mce4F, which had 3,453, 1817, 4896, and 5168 reads in glycerol respectively, and 302, 61, 180 and 32 reads in cholesterol.

Several of the genes identified as differentially essential actually contain read-counts that are significantly suppressed in one condition compared to the other, indicating a selection against insertions, hence suggesting a fitness cost to the organism. An example of such a gene is Rv3200c, which contains 167 reads in glycerol and 1,755 in cholesterol, despite having a substantial number of TA sites with insertions in both conditions (7 out of 13 in glycerol and 9 out of 13 in cholesterol), thus showing the gene can tolerate insertions in both conditions. The relative suppression in read-counts alone is enough to achieve a q-value of 0, suggesting that it is conditionally essential.

To illustrate the comparative analysis on datasets from a different organism, TRANSIT was used to perform a comparative analysis of TnSeq datasets of *H. influenza*. Gawronski et al. [3] compared two datasets of *H. influenza* grown *in vitro* and one dataset derived from lung samples after passaging through mice. The libraries were relatively sparse, with 39% mean density in vitro and 26% for the lung dataset. Gawronski et al. identified a total of 136 genes necessary for growth in lung using a combination of the log ratio of read-counts between conditions and the insertion density *in vitro*. Using TRANSIT’s comparative analysis, 342 genes are obtained using a *q* < 0.05 cutoff. Out of the 136 genes identified by Gawronski et al., 133 (98%) are identified by TRANSIT as differentially essential.

## Availability and Future Directions

TRANSIT standardizes many of the complex steps (workflow) in processing analysis of TnSeq datasets, and provides a user-friendly interface that facilitates analysis of TnSeq data, particularly for libraries generated using the Himar1 transposon. The current version provides three different methods for identifying essential genes, including a method for comparative analysis of conditional essentiality between different conditions.

TRANSIT is written in the Python programming language, and can run on Linux, Macs, or Windows PCs. TRANSIT requires several Python modules (like Scipy for scientific computation, and wxPython for the user-interface), and these dependencies must also be installed. Installation instructions are provided in the manual.

TRANSIT is an Open-Source software platform that can be extended in future releases to include other analysis methods as they are developed. Source code for TRANSIT is available is distributed under the GNU GPL v3 license, and available at the following GitHub repository: https://github.com/mad-lab/transit. The package includes the Python implementation of TRANSIT and TPP, the *M. tuberculosis* TnSeq data used in this article, and documentation.

## Supporting Information

S1 Data Source code and datasets. Source Code for TRANSIT and TPP, and datasets used to obtain results. Please see the GitHub Repository https://github.com/mad-lab/transit to obtain the latest version of the software.
(GZ) Click here for additional data file.
