# Supplementary material for: TRANSIT - A Software Tool for Himar1 TnSeq Analysis
Source: PLoS Comput Biol. 2015 Oct 8;11(10):e1004401. doi: 10.1371/journal.pcbi.1004401 (PMC4598096; doi:10.1371/journal.pcbi.1004401)
Supplement: S1 Data — Source Code for TRANSIT and TPP, and datasets used to obtain results. Please see the GitHub Repository https://github.com/mad-lab/transit to obtain the latest version of the software. (GZ) [file pcbi.1004401.s001.gz › transit_1.4.0/genomes/genomes.html]

H37Rv.fna H37Rv.prot\_table Stewart Cole's reference *M. tuberculosis* sequence (NC\_000962.2)| H37RvMA2.fna H37RvMA2.prot\_table Sequenced strain from the Sassetti lab, with sequencing errors fixed (Ioerger et al. 2010; http://www.ncbi.nlm.nih.gov/pubmed/20472797).|  |  |  |  |  |  |  |  |  |  |  |  | | --- | --- | --- | --- | --- | --- | --- | --- | --- | --- | --- | --- | | H37RvBD.fna H37RvBD.prot\_table Sequenced by the Broad Institute (NC\_018143.2)|  |  |  |  |  |  |  |  |  | | --- | --- | --- | --- | --- | --- | --- | --- | --- | | BCG.fna BCG.prot\_table Reference genome for *M. bovis* BCG (NC\_008769.1)| mc2\_155\_tamu.fna mc2\_155\_tamu.prot\_table *M. smegmatis* mc2 155 sequenced at TAMU| H37RvBD\_mod3.prot\_table Annotation adjusted for an indel in the version of the H37Rv Broad sequence used in the Sassetti lab (for analyzing old datasets that have been previously processed using this sequence).| |  | | | | | | | | | | | | | | | | | | |
