# Supplementary material for: TRANSIT - A Software Tool for Himar1 TnSeq Analysis
Source: PLoS Comput Biol. 2015 Oct 8;11(10):e1004401. doi: 10.1371/journal.pcbi.1004401 (PMC4598096; doi:10.1371/journal.pcbi.1004401)
Supplement: S1 Data — Source Code for TRANSIT and TPP, and datasets used to obtain results. Please see the GitHub Repository https://github.com/mad-lab/transit to obtain the latest version of the software. (GZ) [file pcbi.1004401.s001.gz › transit_1.4.0/doc/TPP.html]

TPP Documentation


## Tn-Seq Pre-Processor (TPP) Documentation

### Overview

TPP is a software tool for processing raw reads (e.g. .fastq files, *untrimmed*) from an
Tn-Seq experiment, extracting counts of transposon insertions at individual TA
dinucleotides sites in a genome ("read counts", or more specifically "template
counts", see below), and writing this information out in .wig format
suitable for input to TRANSIT (add link?). In addition, TPP
calculates some useful statistics and diagnostics on the dataset.

There are many way to do pre-processing of Tn-Seq datasets, and it can depend
on the the protocol used for Tn-Seq, the conventions used by the sequencing
center, etc. However, TPP is written to accommodate the most common situation
among our collaborating labs. In particular, it is oriented toward the Tn-Seq
protocol developed in the Sassetti lab and described in (Long
et al, 2015), which uses a barcoding system to uniquely identifying reads
from distinct transposon-junction DNA fragments. This allows raw read counts
to be reduced to unique **template counts**, eliminating effects of PCR
bias. The sequencing must be done in paired-end (PE) mode (with a minimum
read-length of around 50 bp). The transposon terminus appears in the prefix
of read1 reads, and barcodes are embedded in read2 reads.

The suffixes of read1 and read2 contain nucleotides from the genomic
region adjacent to the transpsoson insertion. These subsequences must be
mapped into the genome. TPP uses BWA (Burroughs-Wheeler Aligner) to
do this mapping. It is a widely-used tool, but you will have to install it on
your system. Mapping large datasets takes time, on the order of 15 minutes
(depending on many factors), so you will have to be patient.

Subsequent to the BWA mapping step, TPP does a bunch of post-processing
steps. Primarily, it tabulates raw read counts at each TA site in the
reference genome, reduces them to template counts, and writes this out
in .wig format (as input for TRANSIT). It also calculates and reports
some statistics on the dataset which a useful for diagnostic purposes.
These are saved in local file caled ".tn\_stats". The GUI automatically
reads all the .tn\_stats files from previously processed datasets in a
directory and displays them in a table.

The GUI interface is set-up basically as a graphical front-end that
allows you to specify input files and parameters to get a job started.
Once you press START, the graphical window goes away, and the pre-processing
begins, printing out status messages in the original terminal window. You
can also run TPP directly from the command-line with the GUI,
by providing all the inputs via command-line arguments.

TPP has a few optional parameters in the interface. We intend to add
other options in the future, so if you have suggestions, let us know. In
particular, if you have some datasets that requires special processing (such
as if different primer sequences were used for PCR amplification, or a
different barcoding system, or different contaminant sequences to search for,
etc.), we might be able to add some options to deal with this.

### Installation

TPP should work equivalently on Macs, PCs running Windows, or Unix machines.
TPP is fundamentally a python script that has a graphical user interface (GUI)
written in wxPython. Its major dependency is that it calls BWA to map reads.
TPP has the following requirements. If these are not already on your system,
you will have to install them manually.

Requirements:

- python version 2.7- wxPython 3.0.1 (the 'cocoa' version)- BWA version 0.7.12  (can put this directory anywhere; be sure to run 'make' to build bwa executable
      - pre-compiled version for 64-bit Windows)

Since TPP is a python script, there is nothing to compile or 'make'.

### Running TPP

TPP may be run from the command line (e.g. of a terminal window or shell)
by typing:

```
python PATH/src/tpp.py
```

where PATH is the path to the TRANSIT installation directory. This should pop up the GUI window, looking like this...

Note, TPP can process paired-end reads, as well as single-end datasets. (just leave the filename for read2 blank)

The main fields to fill out in the GUI are...

- bwa executable - you'll have to find the path to where the executable is installed- reference genome - this is the sequence in Fasta format against which the
    reads will be mapped- reads1 file - this should be the raw reads file (*untrimmed*) for read1 in FASTQ or FASTA format,
      e.g. DATASET\_NAME\_R1.fastq
      - *Note: you can also supply gzipped files for reads, e.g. \*.fastq.gz*- reads2 file - this should be the raw reads file (*untrimmed*) for
        read2 in FASTQ or FASTA format, e.g. DATASET\_NAME\_R2.fastq
        - *Note: if you leave read2 blank, it will process the dataset as single-ended. Since there are no barcodes, each read will be counted as a unique template.*- prefix to use for filename (for the multiple intermediate files that will
          get generated in the process; when you pick datasets, a temp file name
          will automatically be suggested for you, but you can change it to whatever
          you want)- transposon used - Himar1 is assumed by default, but you can set it
            to Tn5 to process libraries of that type. The main consequences of
            this setting are: 1) the selected transposon determines the nucleotide
            prefix to be recognized in read 1, and 2) for Himar1, reads are
            counted only at TA sites, whereas for Tn5, reads are counted at ALL
            sites in the genome (since it does not have significant sequence
            specificity) and written out in the .counts and .wig files.- max reads - Normally, leave this blank by default, and TPP will process
              all reads. However, if you want to do a quick run on a subset of the data,
              you can select a smaller number. This is mainly for testing purposes.- mismatches - this is for searching for the sequence patterns in reads
                corresponding to the transposon prefix in R1 and the constant adapter sequences
                surrounding the barcode in R2; we suggest using a default value of 1 mismatch

Once you have filled all these fields out, you can press START (or QUIT). At
this point the GUI window will disappear, and the data processing commences in the
original terminal/shell windows. It prints out a lot of information to let
you know what it is doing (and error messages, if anything goes wrong). Many
intermediate files get generated. It takes awhile (like on the order of 15
minutes), most of which is taken up by the mapping-reads step by BWA.

Subsequent to the BWA mapping step, TPP does a bunch of
post-processing steps. Primarily, it tabulates raw read counts at each TA
site in the reference genome, reduces them to template counts, and writes this
out in .wig format (as input for essentiality analysis in TRANSIT). It also
calculates and reports some statistics on the dataset which a useful for
diagnostic purposes. These are saved in local file caled "**.tn\_stats**".
The GUI automatically reads all the .tn\_stats files from previously processed
datasets in a directory and displays them in a table.

TPP uses a local config file called "**tpp.cfg**" to rememeber parameter
settings from run to run. This makes it convenient so that you don't have
to type in things like the path to the BWA executable or reference genome
over and over again. You just have to do it once, and TPP will remember.

**Command-line mode:** TPP may be run on a dataset directly from the
command-line without invoking the user interface (GUI) by providing it
filenames and parameters as command-line arguments.

```
For a list of possible command line arguments, type: python tpp.py -help
usage: python TRANSIT_PATH/src/tpp.py -bwa PATH_TO_EXECUTABLE -ref REF_SEQ -reads1 PATH_TO_FASTQ_OR_FASTA_FILE [-reads2 PATH_TO_FASTQ_OR_FASTA_FILE] -prefix OUTPUT_BASE_FILENAME [-maxreads N] [-tn5]
```

The input arguments and file types are as follows:

-bwa path executable |  |  |  |  |  |  |  |  |  |  |  |  |  |  |  |  |  | | --- | --- | --- | --- | --- | --- | --- | --- | --- | --- | --- | --- | --- | --- | --- | --- | --- | | -ref reference genome sequence FASTA file|  |  |  |  |  |  |  |  |  |  |  |  |  |  | | --- | --- | --- | --- | --- | --- | --- | --- | --- | --- | --- | --- | --- | --- | | -reads1 file of read 1 of paired reads FASTA or FASTQ format (or gzipped)|  |  |  |  |  |  |  |  |  |  |  | | --- | --- | --- | --- | --- | --- | --- | --- | --- | --- | --- | | -reads2 file of read 2 of paired reads (optional for single-end reads) FASTA or FASTQ format (or gzipped)|  |  |  |  |  |  |  |  | | --- | --- | --- | --- | --- | --- | --- | --- | | -prefix base filename to use for output files|  |  |  |  |  |  | | --- | --- | --- | --- | --- | --- | | -maxreads subset of reads to process (optional); if blank, use all|  |  |  |  | | --- | --- | --- | --- | | -mismatches how many to allow when searching reads for sequence patterns|  |  | | --- | --- | | -tn5 process reads as a Tn5 library (Himar1 is assumed by default)| |  | | | | | | | | | | | | | | | | | | | | |

(Note: if you have already run TPP once, the you can leave out the
specification of the path for BWA, and it will automatically take the path
stored in the config file, tpp.cfg. Same for ref, if you always use the
same reference sequence.)

### Overview of Data Processing Procedure

Here is a brief summary of the steps performed in converting raw reads (.fastq
files) into template counts:

1. Convert .fastq files to .fasta format (.reads).

   - Identify reads with the transposon prefix in R1
     . The sequence searched for is
     ACTTATCAGCCAACCTGTTA, which must start between cycles 5 and 10 (inclusive).
     (Note that this ends in the canonical terminus of the Himar1 transposon,
     TGTTA.) The "staggered" position of this sequence is due to insertion a few
     nucleotides of variable length in the primers used in the Tn-Seq sample prep
     protocol (e.g. 4 variants of Sol\_AP1\_57, etc.). The number of mimatches
     allowed in searching reads for the transposon sequence pattern can be adjusted
     as an option in the interface; the default is 1.

     - Extract genomic part of read 1. This is the suffix following the
       transposon sequence pattern above. However, for reads coming from fragments
       shorter than the read length, the adapter might appear at the other end of R1,
       TACCACGACCA. If so, the adapter suffix is stripped off. (These are
       referred to as "truncated" reads, but they can still be mapped into the
       genome just fine by BWA.) The length of the genomic part must be at least 20 bp.

       - Extract barcodes from read 2. Read 2 is searched for
         GATGGCCGGTGGATTTGTGnnnnnnnnnnTGGTCGTGGTAT". The length of the barcode is
         typically 10 bp, but can be varaible, and must be between 5-15 bp.

         - Extract genomic portions of read 2. This is the part following
           TGGTCGTGGTAT.... It is often the whole suffix of the read. However, if the
           read comes from a short DNA fragment that is shorter than the read length,
           the adapter on the other end might appear, in which case it is stripped off
           and the nucleotides in the middle representing the genomic insert,
           TGGTCGTGGTATxxxxxxxTAACAGGTTGGCTGATAAG. The insert must be at least 20 bp
           long (inserts shorter than this are discarded, as they might map to spurious
           locations in the genome).

           - Map genomic parts of R1 and R2 into the genome using BWA. Mismatches
             are allowed, but indels are ignored. No trimming is performed. BWA is run in
             'sampe' mode (treating reads as pairs). Both reads of a pair must map (on
             opposite strands) to be counted.

             - Count the reads mapping to each TA site in the reference genome.

               - Reduce raw read counts to unique template counts. Group reads by barcode
                 AND mapping location of read 2 (aka fragment "endpoints").

                 - Output template counts at each TA site in a .wig file.

                   - Calculate statistics like insertion\_density and NZ\_mean. Look for the
                     site with the max template count. Look for reads matching the primer
                     or vector sequences.

**Statistics**

Here is an explanation of the statistics that are saved in the .tn\_stats file
and displayed in the table in the GUI. For convenience, all the statistics
are written out on one line with tab-separation at the of the .tn\_stats file,
to make it easy to add it as a row in a spreadsheet, as some people like to do
to track multiple datasets.

total\_reads total number of reads in the original .fastq/.fasta files|  |  |  |  |  |  |  |  |  |  |  |  |  |  |  |  |  |  |  |  |  |  |  |  |  |  |  |  |  |  |  |  |  |  |  |  | | --- | --- | --- | --- | --- | --- | --- | --- | --- | --- | --- | --- | --- | --- | --- | --- | --- | --- | --- | --- | --- | --- | --- | --- | --- | --- | --- | --- | --- | --- | --- | --- | --- | --- | --- | --- | | truncated\_reads reads representing DNA fragments shorter than the read length; adapter appears at end of read 1 and is stripped for mapping| TGTTA\_reads number of reads with a proper transposon prefix (ending in TGTTA in read1) | |  reads1\_mapped number of R1 mappped into genome (independent of R2)|  |  |  |  |  |  |  |  |  |  |  |  |  |  |  |  |  |  |  |  |  |  |  |  |  |  |  |  |  |  | | --- | --- | --- | --- | --- | --- | --- | --- | --- | --- | --- | --- | --- | --- | --- | --- | --- | --- | --- | --- | --- | --- | --- | --- | --- | --- | --- | --- | --- | --- | | reads2\_mapped number of R2 mappped into genome (independent of R1)|  |  |  |  |  |  |  |  |  |  |  |  |  |  |  |  |  |  |  |  |  |  |  |  |  |  |  |  | | --- | --- | --- | --- | --- | --- | --- | --- | --- | --- | --- | --- | --- | --- | --- | --- | --- | --- | --- | --- | --- | --- | --- | --- | --- | --- | --- | --- | | **mapped\_reads** number of reads which mapped into the genome (requiring both read1 and read2 to map)|  |  |  |  |  |  |  |  |  |  |  |  |  |  |  |  |  |  |  |  |  |  |  |  |  |  | | --- | --- | --- | --- | --- | --- | --- | --- | --- | --- | --- | --- | --- | --- | --- | --- | --- | --- | --- | --- | --- | --- | --- | --- | --- | --- | | read\_count total reads mapping to TA sites (mapped reads excluding those mapping to non-TA sites)|  |  |  |  |  |  |  |  |  |  |  |  |  |  |  |  |  |  |  |  |  |  |  |  | | --- | --- | --- | --- | --- | --- | --- | --- | --- | --- | --- | --- | --- | --- | --- | --- | --- | --- | --- | --- | --- | --- | --- | --- | | template\_count reduction of mapped reads to unique templates using barcodes|  |  |  |  |  |  |  |  |  |  |  |  |  |  |  |  |  |  |  |  |  |  | | --- | --- | --- | --- | --- | --- | --- | --- | --- | --- | --- | --- | --- | --- | --- | --- | --- | --- | --- | --- | --- | --- | | template\_ratio read\_count / template\_count|  |  |  |  |  |  |  |  |  |  |  |  |  |  |  |  |  |  |  |  | | --- | --- | --- | --- | --- | --- | --- | --- | --- | --- | --- | --- | --- | --- | --- | --- | --- | --- | --- | --- | | TA\_sites total number of TA dinucleotides in the genome|  |  |  |  |  |  |  |  |  |  |  |  |  |  |  |  |  |  | | --- | --- | --- | --- | --- | --- | --- | --- | --- | --- | --- | --- | --- | --- | --- | --- | --- | --- | | TAs\_hit number of TA sites with at least 1 insertion|  |  |  |  |  |  |  |  |  |  |  |  |  |  |  |  | | --- | --- | --- | --- | --- | --- | --- | --- | --- | --- | --- | --- | --- | --- | --- | --- | | **insertion\_density** TAs\_hit / TA\_sites|  |  |  |  |  |  |  |  |  |  |  |  |  |  | | --- | --- | --- | --- | --- | --- | --- | --- | --- | --- | --- | --- | --- | --- | | max\_count the maximum number of templates observed at any TA site|  |  |  |  |  |  |  |  |  |  |  |  | | --- | --- | --- | --- | --- | --- | --- | --- | --- | --- | --- | --- | | max\_site the coordinate of the site where the max count occurs|  |  |  |  |  |  |  |  |  |  | | --- | --- | --- | --- | --- | --- | --- | --- | --- | --- | | **NZ\_mean** mean template count over non-zero TA sites|  |  |  |  |  |  |  |  | | --- | --- | --- | --- | --- | --- | --- | --- | | FR\_corr correlation between template counts on Fwd strand versus Rev strand|  |  |  |  |  |  | | --- | --- | --- | --- | --- | --- | | BC\_corr correlation between read counts and template counts over non-zero sites|  |  |  |  | | --- | --- | --- | --- | | primer\_matches how many reads match the primer sequence (primer-dimer problem in sample prep)|  |  | | --- | --- | | vector\_matches how many reads match the phage sequence (transposon vector) used in Tn mutant library construction| |  | | | | | | | | | | | | | | | | | | | | | | | | | | | | | | | | | | | | |

Here is an example of a .tn\_stats file:

```
# title: Tn-Seq Pre-Processor
# date: 02/18/2015 09:36:04
# command: python /pacific/home/ioerger/transit/tpp.py
# read1: /pacific/HomeFrozen/Tn-H37Rv-in-vitro_R1.fastq
# read2: /pacific/HomeFrozen/Tn-H37Rv-in-vitro_R2.fastq
# ref_genome: /pacific/home/ioerger/transit/genomes/H37Rv.fna
# total_reads 1301968 (read pairs)
# truncated_reads 26000 (fragments shorter than the read length; ADAP2 appears in read1)
# TGTTA_reads 1090333 (reads with valid Tn prefix, and insert size>20bp)
# reads1_mapped 1016860
# reads2_mapped 427481
# mapped_reads 413251 (both R1 and R2 map into genome)
# read_count 400069 (TA sites only)
# template_count 211128
# template_ratio 1.89 (reads per template)
# TA_sites 74605
# TAs_hit 21072
# density 0.282
# max_count 2306 (among templates)
# max_site 212278 (coordinate)
# NZ_mean 10.0 (among templates)
# FR_corr 0.917 (Fwd templates vs. Rev templates)
# BC_corr 0.965 (reads vs. templates, summed over both strands)
# primer_matches: 78 reads contain CTAGAGGGCCCAATTCGCCCTATAGTGAGT
# vector_matches: 2 reads contain CTAGACCGTCCAGTCTGGCAGGCCGGAAAC
/pacific/HomeFrozen/Tn-H37Rv-in-vitro_R1.fastq	/pacific/HomeFrozen/Tn-H37Rv-in-vitro_R2.fastq	1301968	1090333	1016860	427481	413251	1016860	427481	400069	211128	1.89491209124	74605	21072	2306	212278	10.0193621868	0.917104229568	0.96542310842	78	2
```

**Interpretation:**
To assess the quality of a dataset, I would recommend starting by looking at 3
primary statistics:

1. **mapped reads**: should be on the order of several million mapped\_reads;
   if there is a significant reduction from total\_reads, look at
   reads1\_mapped and reads2\_mapped and truncated\_reads to figure what
   might have gone wrong; you might try allowing 2 mismatches- **primer/vector\_matches**: check whether a lot of the reads
     might be matching the primer or vector sequences; if they match the
     vector, it suggests your library still has phage contamination from
     the original infection; if there are a lot of primer reads, these
     probably represent "primer-dimers", which could be reduced by
     inproving fragment size selection during sample prep.- **insertion density**: good libraries should have insertions at ≥ ~35% of TA
       sites for statistical analysis- **NZ\_mean**: good datasets should have a mean of around 50 templates per site
         for sufficient dynamic range

If something doesn't look right, the other statistics might be helpful in
figuring out what went wrong. If you see a significant reduction in reads, it
could be due to some poor sequencing cycles, or using the wrong reference
genome, or a contaminant of some type. Some attrition is to be expected (loss
of maybe 10-40% of the reads). The last 2 statistics indicate 2 common cases:
how many reads match the primer or vector sequences. Hopefully these counts
will be low, but if they represent a large fraction of your reads, it could
mean you have a problem with your sample prep protocol or Tn mutant library,
respectively.

### Comments or Questions?

TPP was developed by Thomas
R. Ioerger at Texas A&M University. If you have any comments or
questions, please feel free to send me an email at:
ioerger@cs.tamu.edu
