# Supplementary material for: TRANSIT - A Software Tool for Himar1 TnSeq Analysis
Source: PLoS Comput Biol. 2015 Oct 8;11(10):e1004401. doi: 10.1371/journal.pcbi.1004401 (PMC4598096; doi:10.1371/journal.pcbi.1004401)
Supplement: S1 Data — Source Code for TRANSIT and TPP, and datasets used to obtain results. Please see the GitHub Repository https://github.com/mad-lab/transit to obtain the latest version of the software. (GZ) [file pcbi.1004401.s001.gz › transit_1.4.0/doc/transit.html]

TRANSIT documentation


# TRANSIT v1.4.0 Documentation

  

## Table of Contents

- Overview- Installation
    - Detailed Instructions: Linux- Detailed Instructions: OSX- Detailed Instructions: Windows- Running TRANSIT
      - GUI Mode- Command line Mode- Analysis Methods
        - Gumbel- HMM- Re-sampling- Tutorial: Comparative Analysis - Glycerol vs Cholesterol
          - Run TRANSIT- Add annotation file- Adding the control datasets- Visualizing read counts- Scatter plot- Adding the experimental datasets- Essentiality analysis using the Gumbel method- Essentiality analysis using the HMM method- Comparative analysis using Re-sampling- Viewing resampling results- Tutorial: Export Reads to IGV
            - Adding the annotation file- Add .wig datasets- Export to IGV

---

## Overview

- This is a software that can be used to analyze Tn-Seq datasets.
  It includes various statistical calculations of essentiality of genes or genomic regions (including conditional essentiality between 2 conditions). These methods were developed and tested as a collaboration between the Sassetti lab (UMass) and the Ioerger lab (Texas A&M).
    
    

    
    
  - TRANSIT assumes you have already done pre-processing of raw sequencing files (.fastq) and extracted read counts (at each TA dinucleotide). Actually, the current protocol utilizes internal barcodes that can be used to reduce raw read counts to unique template counts, and this this is the intended input to TRANSIT. The input for TRANSIT consists of .wig files, which simply list the coordinate of each TA sites in the genome and the number of templates observed.- There are various methods available for pre-processing (converting .fastq files to .wig files). You might have your own scripts (if so, massage the data into .wig format), or you might get the scripts used in the Sassetti lab. For convenience, we are including a separate tool called TPP (Tn-Seq Pre-Processor) with this distribution that encodes the way we process .fastq files in the Ioerger lab. It's a complicated process with many steps (removing transposon prefixes of reads, mapping into genome, identifying barcodes and reducing read counts to template counts).- Most of the analysis methods in TRANSIT require an **annotation** to know the gene coordinates and names. This is the top file input in the GUI window. The annotation has to be in a somewhat non-standard format called a ".prot\_table". If you know what you are doing, it is easy to convert annotations for other organsims into .prot\_table format. But for convenience, we are distributing the prot\_tables for 3 common versions of the H37Rv genome: H37Rv.prot\_table (NC\_000962.2, from Stewart Cole), H37RvMA2.prot\_table (sequenced version from the Sassetti lab), and H37RvBD.prot\_table (sequenced by the Broad Institute). All of these are slightly different, and it is **critical** that you use the same annotation file as the reference genome sequence used for mapping the reads (during pre-processing).- There are 2 main types of essentiality analyses: individaul, comaparative. In individual analysis, the goal is to distinguish essential vs. non-essential in a single growth condition, and to assess the statistical significance of these calls. Two methods for this are the Gumbel method and the HMM. They are computationally distinct. The Gumbel method is looking for significant stretches of TA sites lacking insertions, whereas the HMM looks for regions where the mean read count is locally suppresed or increased. The HMM can detect 'growth-advantaged' and 'growth-defect' regions. The HMM is also a bit more robust on low-density datasets (insertion density 20-30%). But both methods have their merits and are complementary. For compararative analysis, TRANSIT uses 're-sampling', which is analogous to a permutation test, to determine if the sum of read counts differs significantly between two conditions. Hence this can be used to identify conditionally essential regions and quantify the statistical significance.- TRANSIT has been designed to handle multiple replicates. If you have two or more replicate dataset of the same libarary selected in the same condition, you can provide them, and more of the computational methods will do something reasonable with them.- For those methods that generate p-values, we often also calculate adjusted p-value (or 'q-values') which are corrected for multiple tests typically the Benjamini-Hochberg procedure. A typical threshold for significance would be q<0.05 (not p<0.05).- It is important to understand the GUI model that TRANSIT uses It allows you to load up datasets (.wig files), select them, choose an analysis method, set parameters, and start the computation. It will generate **output files** in your local directory with the results. These files can then be loaded into the interface and browser with custom displays and graphs. The interface has 3 main windows or sections: 'Control Samples', 'Experimental Samples', 'Results Files.' The first two are for loading input files ('Control Samples' would be like replicate datasets from a reference condition, like in vitro, rich media, etc.; 'Experimental Samples' would be where you would load replicates for a comparative conditions, like in vivo, or minimmal media, or low-iron, etc.) The 'Results Files' section is initially empty, but after a computation finishes, it will automatically be populated with the corresponding output file. See the 'Tutorial' section below in this documentation for an illustraion of the overall process for a typical work-flow.- TRANSIT incorporates many interesting ways of looking at your data.
                  - Track view shows you a visual representation of the read counts at each site at a locus of interest (for selected datasets) somewhat like IGV.
                      
                      

                      
                      
                    - Scatter plots can show the correlation of counts between 2 datasets.
                        
                        

                        
                        
                      - Volcano plots can be used to visualize the results of resampling and assess the distribution between over- and under-represented genes in condition B vs. condition A. In addition you can look at histogram of the re-sample distributions for each gene.- Most of the methods take a few minutes to run. (it depends on parameters, CPU clock speed, etc., but the point is, a) these calculations are complex and not instaneous, but b) we have tried to implement it so that they don't take hours)- Note: in this version of TRANSIT, most of the methods are oriented toward gene-level analysis. There are methods for analyzing essentiality of arbitrary genomic regions (e.g. sliding windows, HMMs...). We plan to incorporate some of these in future versions.

---

## Installation

TRANSIT is python-based. You must have python installed (installed by default on most systems). In addition, TRANSIT relies on some python packages/libraries/modules that you might need to install. Below are the list of requirements:

### Requirements

The following libraries/modules are required to run TRANSIT:
  

- Python 2.7- Numpy 1.6.1+- Scipy 0.14.0+- matplotlib 1.1.1+- wxpython 2.8.0+ (for Mac OSX, use the **cocoa** version of wxPython)- PIL (Python Imaging Library) or Pillow.

  
Generally, these requirements are install using the appropriate methods for your operating system, i.e. apt-get or yum for unix machines, pip or easy\_install for OSX, or binary installers on Windows. Below more detailed instructions are provided.
  

### Detailed Instructions: Linux

Most of the requirements are available in default package sources in most Linux distributions. The following commands will install python, numpy, scipy, matplotlib on the Ubuntu or Fedora Linux distributions:

```
#Ubuntu:
sudo apt-get install python python-numpy python-scipy python-matplotlib python-wxgtk3.0

#Fedora:
sudo yum install python numpy scipy python-matplotlib python-wxgtk3.0
```

  
The final requirement left to install is Pillow. First you need install *pip* which simplifies the process of installing certain python modules like Pillow:
  

```
#Ubuntu:
sudo apt-get install pip

#Fedora:
sudo yum install pip
```

  
Next, using pip you must have a clean installation of Pillow, and the desired libraries. You can achieve this through the following commands:

```
#Ubuntu:
pip uninstall pillow
pip uninstall Pillow
sudo apt-get install libjpeg-dev zlib1g-dev
pip install -I Pillow

#Fedora:
pip uninstall pillow
pip uninstall Pillow
sudo yum install install libjpeg-dev zlib1g-dev
pip install -I Pillow
```

Optional: If you will be using the pre-processor, TPP, you will also need to install BWA. Download the source files:

```
http://sourceforge.net/projects/bio-bwa/files/
```

  
Extract the files:

```
tar -xvjf bwa-0.7.12.tar.bz2
```

  
Go to the directory with the extracted source-code, and run make to create the executable files:

```
cd bwa-0.7.12
make
```

  

### Detailed Instructions: OSX

First, download and install the latest Python 2.7.x installation file from the official python website:
  

```
http://www.python.org/downloads/
```

  
Next make sure you have pip installed. Pip can be installed through easy\_install, which should come with OSX:

```
sudo easy_install pip
```

  
Next install numpy, scipy, and matplotlib and pillow using pip:

```
sudo pip install numpy
sudo pip install scipy
sudo pip install matplotlib
sudo pip install pillow
```

  
Download and install the OSX binary of wxpython (cocoa version) for python 2.7:

```
http://downloads.sourceforge.net/wxpython/wxPython3.0-osx-3.0.2.0-cocoa-py2.7.dmg
```

Optional: If you will be using the pre-processor, TPP, you will also need to install BWA
. Download the source files:

```
http://sourceforge.net/projects/bio-bwa/files/
```

  
Extract the files:

```
tar -xvjf bwa-0.7.12.tar.bz2
```

  
Go to the directory with the extracted source-code, and run make to create the executable files:

```
cd bwa-0.7.12
make
```

  

### Detailed Instructions: Windows

First, download and install the latest Python 2.7.x installation file from the official python website:
  

```
http://www.python.org/downloads/
```

  
Next, you will need to install pip. If you are using python 2.7.9+ then pip will come pre-installed and included in the default script directory (i.e. *C:\Python27\Scripts*). If you are using python 2.7.8 or older, you will need to manually install pip by downloading and running the get-pip.py script:
  

```
python.exe get-pip.py
```

  
Make sure that "wheel" is installed. This is necessary to allow you to install .whl (wheel) files:

```
pip.exe install wheel
```

  
Download the .whl files for all the requirements (Note: Make sure to choose the files that match your Windows version i.e. 32/64 bit)

- ```
  numpy-1.9.2+mkl-cp27-none-win_amd64.whl [32 bit]
  ```

  - ```
    scipy-0.15.1-cp27-none-win_amd64.whl [32 bit]
    ```

    - ```
      matplotlib-1.4.3-cp27-none-win_amd64.whl [32 bit]
      ```

      - ```
        Pillow-2.8.1-cp27-none-win_amd64.whl [32 bit]
        ```

        - ```
          wxPython-3.0.2.0-cp27-none-win_amd64.whl [32 bit]
          ```

          - ```
            wxPython_common-3.0.2.0-py2-none-any.whl [32 bit]
            ```

```
Source: These files were obtained from the Unofficial Windows Binaries for Python Extension Packages
by Christoph Gohlke, Laboratory for Fluorescence Dynamics, University of California, Irvine.
```

  
Finally, install the files using pip:

```
pip.exe install numpy-1.9.2+mkl-cp27-none-win_amd64.whl
pip.exe install scipy-0.15.1-cp27-none-win_amd64.whl
pip.exe install matplotlib-1.4.3-cp27-none-win_amd64.whl
pip.exe install Pillow-2.8.1-cp27-none-win_amd64.whl
pip.exe install wxPython-3.0.2.0-cp27-none-win_amd64.whl
pip.exe install wxPython_common-3.0.2.0-py2-none-any.whl
```

  
Optional: If you will be using the pre-processor, TPP, you will also need to install BWA. We provide a windows executable (.exe) for Windows 64 bit:
  

bwa-0.7.12\_windows.zip
  
  


---

  

## Running TRANSIT

### GUI Mode

To run TRANSIT in GUI mode (should be the same on Linux, Windows and MacOS), from the command line run:

```
python PATH/src/transit.py
```

where PATH is the path to the TRANSIT installation directory. You might be able to double-click on icon for transit.py, if your OS associates .py files with python and automatically runs them.
Note, because TRANSIT has a graphical user interface, if you are trying to run TRANSIT across a network, for example, running on a unix server but displaying on a desktop machine, you will probably need to use 'ssh -Y' and a local X11 client (like Xming or Cygwin/X on PCs).

### Command line Mode

TRANSIT can also be run from the command line, without the GUI interface. This is convenient if you want to run many analyses in batch, as you can write a script that automatically runs that automatically runs TRANSIT from the command line.
  
TRANSIT expects the user to specify which analysis method they wish to run. The user can choose from "gumbel", "hmm", or "resampling". By choosing a method, and adding the "-h" flag, you will get a list of all the necessary parameters and optional flags for the chosen method:

```
python PATH/src/transit.py gumbel -h
```

  

#### Gumbel

To run the Gumbel analysis from the command line, type "python PATH/src/transit.py gumbel" followed by the following arguments:

Argument | Type | Description | Default | Example || annotation | Required | Path to annotation file in .prot\_table format |  | genomes/H37Rv.prot\_table |
| control\_files | Required | Comma-separated list of paths to the \*.wig replicate datasets |  | data/glycerol\_reads\_rep1.wig,data/glycerol\_reads\_rep2.wig |
| output\_file | Required | Name of the output file with the results. |  | results/gumbel\_glycerol.dat |
| -s SAMPLES | Optional | Number of samples to take. | 10000 | -s 20000 |
| -m MINREAD | Optional | Smallest read-count considered to be an insertion. | 1 | -m 2 |
| -b BURNIN | Optional | Burn in period, Skips this number of samples before getting estimates. See documentation. | 500 | -b 100 |
| -t TRIM | Optional | Number of samples to trim. See documentation. | 1 | -t 2 |
| -r REP | Optional | How to handle replicates read-counts: 'Sum' or 'Mean'. | Sum | -r Mean |
| -iN IGNOREN | Optional | Ignore TAs occuring at X% of the N terminus. | 5 | -iN 0 |
| -iC IGNOREC | Optional | Ignore TAs occuring at X% of the C terminus. | 5 | -iC 10 |

Example Gumbel command:

```
python PATH/src/transit.py gumbel genomes/H37Rv.prot_table data/glycerol_reads_rep1.wig,data/glycerol_reads_rep2.wig test_console_gumbel.dat -s 20000 -b 1000
```

  

#### HMM

To run the HMM analysis from the command line, type "python PATH/src/transit.py hmm" followed by the following arguments:

Argument | Type | Description | Default | Example || annotation | Required | Path to annotation file in .prot\_table format |  | genomes/H37Rv.prot\_table |
| control\_files | Required | Comma-separated list of paths to the \*.wig replicate datasets |  | data/glycerol\_reads\_rep1.wig,data/glycerol\_reads\_rep2.wig |
| output\_file | Required | Name of the output file with the results. |  | results/gumbel\_glycerol.dat |
| -r REP | Optional | How to handle replicates read-counts: 'Sum' or 'Mean'. | Sum | -r Mean |
| -iN IGNOREN | Optional | Ignore TAs occuring at X% of the N terminus. | 5 | -iN 0 |
| -iC IGNOREC | Optional | Ignore TAs occuring at X% of the C terminus. | 5 | -iC 10 |

Example HMM command:

```
python PATH/src/transit.py hmm genomes/H37Rv.prot_table data/glycerol_reads_rep1.wig,data/glycerol_reads_rep2.wig test_console_hmm.dat -r Sum
```

  

#### Resampling

To run the Resampling analysis from the command line, type "python PATH/src/transit.py resampling" followed by the following arguments:

Argument | Type | Description | Default | Example || annotation | Required | Path to annotation file in .prot\_table format |  | genomes/H37Rv.prot\_table |
| control\_files | Required | Comma-separated list of paths to the \*.wig replicate datasets for the control condition |  | data/glycerol\_reads\_rep1.wig,data/glycerol\_reads\_rep2.wig |
| exp\_files | Required | Comma-separated list of paths to the \*.wig replicate datasets for the experimental condition |  | data/cholesterol\_reads\_rep1.wig,data/cholesterol\_reads\_rep2.wig |
| output\_file | Required | Name of the output file with the results. |  | results/gumbel\_glycerol.dat |
| -s SAMPLES | Optional | Number of permutations performed. | 10000 | -s 5000 |
| -H | Optional | Creates histograms of the permutations for all genes. | Not set | -H |
| -a | Optional | Performs adaptive appoximation to resampling. | Not set | -a |
| -N | Optional | Select which normalizing procedure to use. Can choose between 'nzmean', 'totreads', 'zinfnb', and 'nonorm'. See the parameters section for the Re-sampling method for a description of these normalization options. | nzmean | -N nzmean |
| -iN IGNOREN | Optional | Ignore TAs occuring at X% of the N terminus. | 5 | -iN 0 |
| -iC IGNOREC | Optional | Ignore TAs occuring at X% of the C terminus. | 5 | -iC 10 |

Example Resampling command:

```
python PATH/src/transit.py resampling genomes/H37Rv.prot_table data/glycerol_reads_rep1.wig,data/glycerol_reads_rep2.wig data/cholesterol_reads_rep1.wig,data/cholesterol_reads_rep2.wig,data/cholesterol_reads_rep3.wig test_console_resampling.dat -H -s 10000 -N nzmean
```

  
  
  


---

## Analysis Methods

### Gumbel

#### What does it do?

The Gumbel can be used to determine which genes are essential in a single condition. It does a gene-by-gene analysis of the insertions at TA sites with each gene, makes a call based on the longest consecutive sequence of TA sites without insertion in the genes, calculates the probabily of this using a Baysian model.

#### How does it work?

For a formal description of how this method works, see our paper:
  
DeJesus, M.A., Zhang, Y.J., Sassettti, C.M., Rubin, E.J., Sacchettini,
J.C., and Ioerger,
T.R. (2013).   
Bayesian analysis of gene essentiality based on sequencing of transposon insertion libraries. *Bioinformatics*, 29(6):695-703.

#### Parameters

- **Samples:** Gumbel uses Metropolis-Hastings (MH) to generate samples of posterior distributions. The default setting is to run the simulation for 10,000 iterations. This is usually enough to assure convergence of the sampler and to provide accurate estimates of posterior probabilities. Less iterations may work, but at the risk of lower accuracy.- **Burn-In:** Because the MH sampler many not have stabalized in the first few iterations, a "burn-in" period is defined. Samples obtained in this "burn-in" period are discarded, and do not count towards estimates.- **Trim:** The MH sampler produces Markov samples that are correlated. This parameter dictates how many samples must be attempted for every sampled obtained. Increasing this parameter will decrease the auto-correlation, at the cost of dramatically increasing the run-time. For most situations, this parameter should be left at the default of "1".- **Minimum Read:** The minimum read count that is considered a true read. Because the Gumbel method depends on determining gaps of TA sites lacking insertions, it may be suceptible to spurious reads (e.g. errors). The default value of 1 will consider all reads as true reads. A value of 2, for example, will ignore read counts of 1.- **Replicates:** Determines how to deal with replicates by averaging the read-counts or suming read counts accross datasets. This should not have an affect for the Gumbel method, aside from potentially affecting spurious reads.

#### Outputs and diagnostics

The Gumbel method generates a tab-seperated output file at the location chosen by the user. This file will automatically be loaded into the Results Files section of the GUI, allowing you to display it as a table. Alternatively, the file can be opened in a spreadsheet software like Excel as a tab-separated file. The columns of the output file are defined as follows:

| Column Header | Column Definition |
| --- | --- |
| ORF | Gene ID. |
| Name | Name of the gene. |
| Description | Gene description. |
| k | Number of Transposon Insertions Observed within the ORF. |
| n | Total Number of TA dinucleotides within the ORF. |
| r | Length of the Maximum Run of Non-Insertions observed. |
| s | Span of nucleotidies for the Maximum Run of Non-Insertions. |
| zbar | Posterior Probability of Essentiality. |
| Call | Essentiality call for the gene. Depends on FDR corrected thresholds. E=Essential U=Uncertain, NE=Non-Essential, S=too short |

  
  
Note: Technically, Bayesian models are used to calculate posterior probabilities, not p-values (which is a concept associated with the
frequentist framework). However, we have implemented a method for computing the approximate false-discovery rate (FDR) that serves a
similar purpose. This determines a threshold for significance on the posterior probabilities that is corrected for multiple tests. The actual thresholds used are reported in the headers of the output file (and are near 1 for essentials and near 0 for non-essentials). There can be many genes that score between the two thresholds (t1 < zbar < t2). This reflects intrinsic uncertainty associated with either low read counts, sparse insertion density, or small genes.
If the insertion\_density is too low (< ~30%), the method may not work as well, and might indicate an unusually large number of Uncertain or Essential genes.

#### Run-time

The Gumbel method takes on the order of 10 minutes for 10,000 samples. Run-time is linearly proportional to the 'samples' parameter, or length of MH sampling trajectory.
Other notes: Gumbel can be run on multiple replicates; replicate datasets will be automatically merged.

---

### HMM

#### What does it do?

The HMM method can be used to determine the essentiality of the entire genome, as opposed to gene-level analysis of the other methods. It is capable of identifying regions that have unusually high or unusually low read counts (i.e. growth advantage or growth defect regions), in addition to the more common categories of essential and non-essential.

#### How does it work?

For a formal description of how this method works, see our paper:
  
DeJesus, M.A., Ioerger, T.R. A Hidden Markov Model for identifying essential and growth-defect regions in bacterial genomes from transposon insertion sequencing data. *BMC Bioinformatics.* 2013. 14:303

#### Parameters

The HMM method automatically estimates the necessary statistical parameters from the datasets. You can change how the method handles replicate datasets:

- **Replicates:** Determines how the HMM deals with replicate datasets by either averaging the read-counts or suming read counts accross datasets. For regular datasets (i.e. mean-read count > 100) the recommended setting is to average read-counts together. For sparse datasets, it suming read-counts may produce more accurate results.

#### Output and Diagnostics

The HMM method outputs two files. The first file provides the most likely assignment of states for all the TA sites in the genome. Sites can belong to one of the following states: "E" (Essential), "GD" (Growth-Defect), "NE" (Non-Essential), or "GA" (Growth-Advantage). In addition, the output includes the probability of the particular site belonging to the given state. The columns of this file are defined as follows:
  
  

| Column # | Column Definition |
| --- | --- |
| 1 | Coordinate of TA site |
| 2 | Observed Read Counts |
| 3 | Probability for ES state |
| 4 | Probability for GD state |
| 5 | Probability for NE state |
| 6 | Probability for GA state |
| 7 | State Classification (ES = Essential, GD = Growth Defect, NE = Non-Essential, GA = Growth-Defect) |
| 8 | Gene(s) that share(s) the TA site. |

  
  
The second file provides a gene-level classification for all the genes in the genome. Genes are classified as "E" (Essential), "GD" (Growth-Defect), "NE" (Non-Essential), or "GA" (Growth-Advantage) depending on the number of sites within the gene that belong to those states.
  
  

| Column Header | Column Definition |
| --- | --- |
| Orf | Gene ID |
| Name | Gene Name |
| Desc | Gene Description |
| N | Number of TA sites |
| n0 | Number of sites labeled ES (Essential) |
| n1 | Number of sites labeled GD (Growth-Defect) |
| n2 | Number of sites labeled NE (Non-Essential) |
| n3 | Number of sites labeled GA (Growth-Advantage) |
| Avg. Insertions | Mean insertion rate within the gene |
| Avg. Reads | Mean read count within the gene |
| State Call | State Classification (ES = Essential, GD = Growth Defect, NE = Non-Essential, GA = Growth-Defect) |

  
  
Note: Libraries that are too sparse (e.g. < 30%) or which contain very low read-counts may be problematic for the HMM method, causing it to label too many Growth-Defect genes.

#### Run-time

The HMM method takes less than 10 minutes to complete. The parameters of the method should not affect the running-time.
  
  
  
  


---

### Re-sampling

#### What does it do?

The re-sampling method is a comparative analysis the allows that can be used to determine conditional essentiality of genes. It is based on a permutation test, and is capable of determining read-counts that are significantly different across conditions.

#### How does it work?

This technique has yet to be formally published in the context of differential essentiality analysis. Briefly, the read-counts at each genes are determined for each replicate of each condition. The total read-counts in condition A is substracted from the total read counts at condition B, to obtain an observed difference in read counts. The TA sites are then permuted for a given number of "samples". For each one of these permutations, the difference is read-counts is determined. This forms a null disttribution, from which a p-value is calculated for the original, observed difference in read-counts.

#### Parameters

The resampling method is non-parametric, and therefore does not require any parameters governing the distributions or the model. The following parameters are available for the method:

- **Samples:** The number of samples (permutations) to perform. The larger the number of samples, the more resolution the p-values calculated will have, at the expense of longer computation time. The re-sampling method runs on 10,000 samples by default.- **Output Histograms:**Determines whether to output .png images of the histograms obtained from resampling the difference in read-counts.- **Adaptive Resampling:** An optional "adaptive" version of resampling which accelerates the calculation by terminating early for genes which are likely not significant. This dramatically speeds up the computation at the cost of less accurate estimates for those genes that terminate early (i.e. deemed not significant). This option is OFF by default.- **Normalization Method:** Determines which normalization method to use when comparing datasets. Proper normalization is important as it ensures that other sources of variability are not mistakenly treated as real differences.
        - **nzmean**: Normalizes datasets to have the same mean over the non-zero sites.- **totreads**: Normalizes datasets by total read-counts, and scales them to have the same mean over all counts.- **zinfnb**: Fits a zero-inflated negative binomial model, and then divides read-counts by the mean. The zero-inflated negative binomial model will treat some empty sites as belonging to the "true" negative binomial distribution responsible for read-counts while treating the others as "essential" (and thus not influencing its parameters).- **quantile**: Normalizes datasets using the quantile normalization method described by Bolstad et al. (2003). In this normalization procedure, datasets are sorted, an empirical distribution is estimated as the mean across the sorted datasets at each site, and then the original (unsorted) datasets are assigned values from the empirical distribution based on their quantiles.- **nonorm**: No normalization is performed.

#### Output and Diagnostics

The re-sampling method ouputs a tab-delimited file with results for each gene in the genome. P-values are adjusted for multiple comparisons using the Benjamini-Hochberg procedure (called "q-values" or "p-adj."). A typical threshold for conditional essentiality on is q-value < 0.05.

| Column Header | Column Definition |
| --- | --- |
| Orf | Gene ID. |
| Name | Name of the gene. |
| Description | Gene description. |
| N | Number of TA sites in the gene. |
| TAs Hit | Number of TA sites with at least one insertion. |
| Sum Rd 1 | Sum of read counts in condition 1. |
| Sum Rd 2 | Sum of read counts in condition 2. |
| Delta Rd | Difference in the sum of read counts. |
| p-value | P-value calculated by the permutation test. |
| p-adj. | Adjusted p-value controlling for the FDR (Benjamini-Hochberg) |

  
  

#### Run-time

A typical run of the re-sampling method with 10,000 samples will take around 45 minutes (with the histogram option ON). Using the adaptive resampling option, the run-time is reduced to around 10 minutes.

---

## Tutorial: Essentiality Analysis - Glycerol and Cholesterol

To illustrate how TRANSIT works, we are going to go through a tutorial where we analyze datasets of H37Rv *M. tuberculosis* grown on glycerol and cholesterol.

### Run TRANSIT

Navigate to the directory containing the TRANSIT files, and run TRANSIT:

```
python PATH/src/transit.py
```

### Adding the annotation file

Before we can analyze datasets, we need to add an annotation file for the organism corresponding to the desired datasets. Click on the file dialog button, on the top of the TRANSIT window (see image below), and browse and select the appropriate annotation file. Note: Annotation files must be in ".prot\_table" format, described above.
  
  

  
  

### Adding the control datasets

We want to analyze datasets grown in glycerol to those grown in cholesterol. We are choosing the datasets grown in glycerol as the "Control" datasets. To add these, we click on the control sample file dialog (see image below), and select the desired datasets (one by one). In this example, we have two replicates:
  
  

  
  
As we add the datasets they will appear in the table in the Control samples section. This table will provide the following statistics about the datasets that have been loaded so far: Total Number of Reads, Density, Mean Read Count and Maximum Count. These statistics can be used as general diagnostics of the datasets.

### Visualizing read counts

TRANSIT allows us to visualize the read-counts of the datasets we have already loaded. To do this, we must select the desired datasets ("Control+Click") and then click on "View -> Track View" in the menu bar at the top of the TRANSIT window. Only those selected datasets will be displayed:
  
  

  
  
This will open a window that allows that shows a visual representation of the read counts at the TA sites throughout the genome. The scale of the read counts can be set by changing the value of the "Max Read" textbox on the right. We can browse around the genome by clicking on the left and right arrowm, or search for a specific gene with the search text box.
  
  

  
  
This window also allows us to save a .png image of the canvas for future reference if desired (i.e. Save Img button).

### Scatter plot

We can also view a scatter plot of read counts of two selected datasets. To achieve this we select two datasets (using "Control + Clicck") and then clicking on "View -> Scatter Plot" in the menu bar at the top of the TRANSIT window.
  
  

  
  
A new window will pop-up, show a scatter plot of both of the selected datasets. This window contains controls to zoom in and out (magnifying glass), allowing us to focus in on a specific area. This is particularly useful when large outliers may throw off the scale of the scatter plot.
  
  

  
  

### Essentiality analysis with the Gumbel method

Before comparing both conditions against each other, we may want to determine which genes are essential in a specific condition to get an idea of the genes which are required. To do this we can use the Gumbel or the HMM methods, which determine essentiality within one condition.
First we chose the Gumbel method from the drop-down menu on the right hand side of the TRANSIT window:
  
  

  
  
For this particular case we leave the parameters at their default settings as these work with a wide variety of datasets (See above for an explanation of their function). We then click on the "Run Gumbel" button and wait until the analysis finished running. The progress bar will give us information about how much of the analysis is still left.
Once the program finishes, the results file is automatically created (with the name chosen at run-time) and it is automatically added to the Results File section at the bottom of TRANSIT. We can visualize the results by selecting this file from the list, and clicking on the "Display Table" button. This will open a new window with a table of resuls:
  
  

  
  
From this window we can view results, and sort on a specific column (described above) by clicking on a column header. In addition, the top of this window contains a breakdown of the number of essential and non-essential genes found by the Gumbel method. We can see that 675 genes are found to be essential by the Gumbel method (16%), roughly matching expectations that 15% of the genomes is necessary for growth in bacterial organisms. Clicking on the "Zbar" column we can sort the data on the posterior probability of essentiality. If we sort in descending order, we get those genes which are most likely to be essential on the top. Among these are genes like GyrA (DNA gyrase A) and RpoB (DNA-directed polymerase), which are both well-known essential genes, and which are obtain a posterior probability of essentiality of 1.0 (Essential).

### Essentiality analysis with the HMM method

An alternative method for determining essentiality is the HMM method. This method differs from the Gumbel method in that is capable of assessing the essentiality of the entire genome, and is not limited to a gene-level analysis (See above for discussions of the pros and cons of each method). To run the HMM method we select it from the drop-down menu to the right. This automatically displays the available options for the HMM methid. Because the HMM method estimates parameters by examining the datasets, there is no need to set parameters for the model.
One important option provided is how to deal with replicate datasets. Because the glycerol replicates had a mean read-count between 53-85, we decide to sum read-counts together by selecting "Sum" from the drop-down option.
  
  

  
  
Finally we click on the "Run HMM" button, and wait for the method to finish. Once the analysis finishes, two new files will be created and automatically added to the list of files in the Results Files section. One file contains the output of states for each TA site in the genome. The other file contains the analysis for each gene. We can display each of the files be selecting them (individually) and clicking on the "Display Table" button (one at a time).

Like for the Gumbel method, a break down of the states is provided at the top of the table. In the case of glycerol, the HMM analysis classifies 16.3% of the genome as belonging to the "Essential" state, 5.4% belonging to the Growth-Defect state, 77.1% to the Non-Essential state, and 1.2% to the Growth Advantage state. This break down can be used as a diagnostic, to see if the results match our expectations. For example, in datasets with very low read-counts, or very low density, the percentage of Growth-Defect states may be higher (e.g. > 10% ), which could indicate a problem.
  
  

  
  
The HMM sites file contains the state assignments for all the TA sites in the genome. This file is particularly useful to browse for browsing the different types of regions in the genome. We can use this file to see how regions have different impacts on the growth-advantage (or disadvantage) of the organism. For example, the PDIM locus, which is required for virulance in vivo, results in a Growth-Advantage for the organism when disrupted. We can see this in the HMM Sites file by scrolling down to this region (Rv2930-Rv2939) and noticing the large read-counts at these sites, and the how they are labeled "GA".

### Adding the experimental datasets

We now repeat the process we did for control samples, for the experimental datasets that were grown on cholesterol. To add these, we click on the experimental sample file dialog (see image below), and select the desired datasets (one by one). In this example, we have three replicates:
  
  

  
  

### Comparative analysis using Re-sampling

To compare the growth conditions and assess conditional essentiality, we select "Resampling" from the list of methods in the drop-down menu on the right side of the TRANSIT window:
  
  

  
  
This will populate the right side with options specific to the Resampling method. In this case, we choose to proceed with the default settings. However, we could have set a different number of samples for the resampling method or chosen the "Adaptive Resampling" option if we were interested in quicker results. See the description of the method above for more information.

We click on the "Run Resampling" button to start the analysis. This will take several minutes to finish. The progress bar will give us an idea of how much time is left.

### Viewing resampling results

Once TRANSIT finishes running, the results file will automatically be added to the Results Files section at the bottom of the window:
  
  

  
  
This window allows us to track the results files that have been created in this session. From here, we can display a volcano plot of the resampling results by selecting the file from the list and clicking on the "Display Graph" button. This will open a new window containing the figure:
  
  

  
  
To view the actual results, we can open the file in a new window by selecting it from the list and clicking on the "Display Table" button:
  
  

  
  
The newly opened window will display a table of the results. We can sort the results by clicking on the column header. For example, to focus on the genes that are most likely to be conditionally essential between glycerol and cholesterol, we can click on the column header labeled "q-value", which represents p-values that have been adjusted for multiple comparisons.

Sorting q-values in ascending order, we can see those genes which are most likely to be conditionally essential on the top. A typical threshold for significance is < 0.05. We can use "Delta Sum" column to see which conditions had the most read counts in a particular gene. The sign of this value (+/-) lets us know on which condition the gene is essential and which condition it is non-essential. The magnitude lets us know how large the difference is. For example, glycerol kinase (GlpK) is necessary for growth on glycerol but it is not expected to be necessary when grown on another carbon source like cholesterol. We confirm our expectations by noticing that the sum of read counts in glycerol is only 22 reads (normalized), while there are a total of 2119 reads in cholesterol. The difference (2096) is positive, which means it is necessary for growth in glycerol but not cholesterol.

Because we ran the resampling method with the "Histograms" options, we also have the ability to view the histograms of permutation differences for each of the genes by selecting a gene and right clicking:
  
  

  
  
From this menu we can display the histogram, or view the read-counts for that specific gene in Track View:
  
  

  
  

---

## Tutorial: Export Reads to IGV

### Adding the annotation file

Before we can export .wig datasets to IGV format, we need to add an annotation file for the organism. Click on the file dialog button, on the top of the TRANSIT window (see image below), and browse and select the appropriate annotation file. Note: Annotation files must be in ".prot\_table" format, described above:
  
  

  
  

### Add .wig datasets

Next we must choose to add .wig formatted datasets what we wish to export to IGV format. To add these, we click on the control sample file dialog (see image below), and select the desired datasets (one by one). In this example, we have two replicates:
  
  

  
  
As we add the datasets they will appear in the table below.

### Export to IGV

Finally, to export the datasets we click on "Export" in the menu bar at the top of the TRANSIT window, and select the option that matches which datasets we wish to export. Note: Only selected datasets ("Control+Click") will be exported.
