# Supplementary figures and images for: TRANSIT - A Software Tool for Himar1 TnSeq Analysis
Source: PLoS Comput Biol. 2015 Oct 8;11(10):e1004401. doi: 10.1371/journal.pcbi.1004401 (PMC4598096; doi:10.1371/journal.pcbi.1004401)

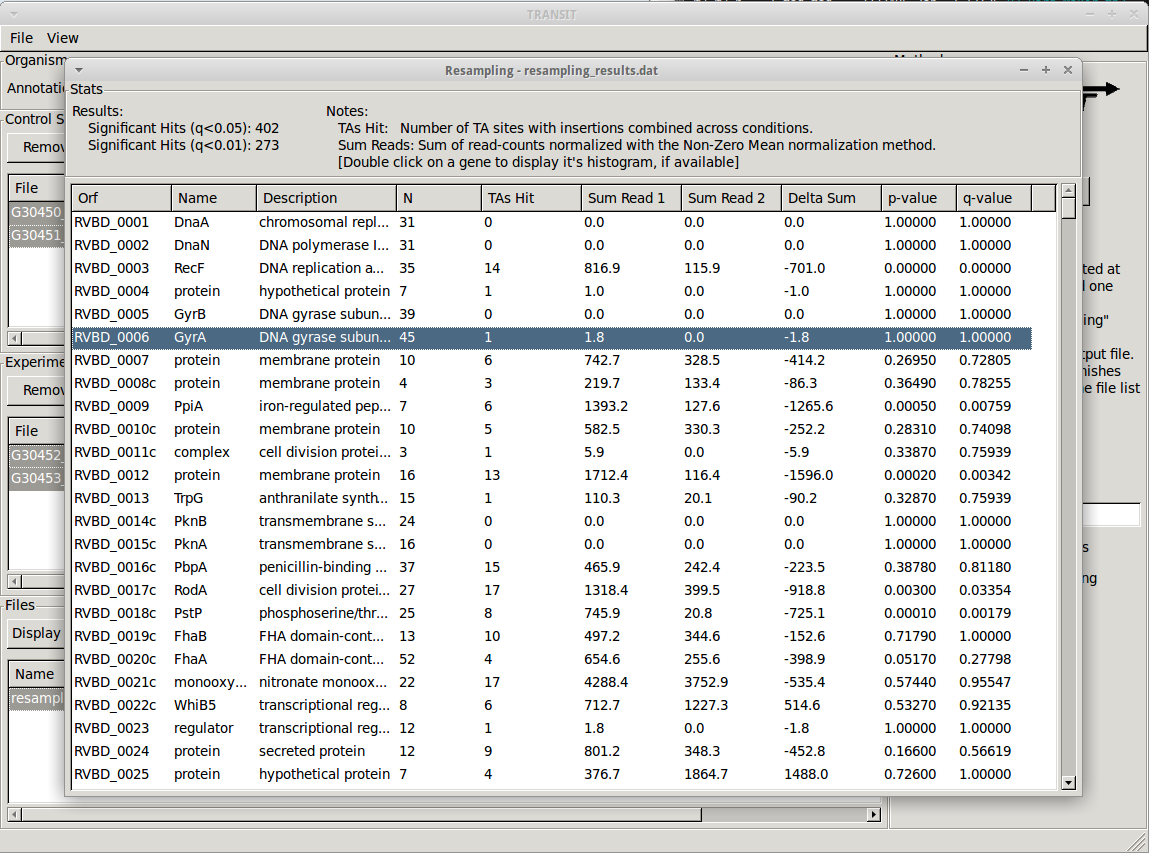

Supplement: S1 Data — Source Code for TRANSIT and TPP, and datasets used to obtain results. Please see the GitHub Repository https://github.com/mad-lab/transit to obtain the latest version of the software. (GZ) [file pcbi.1004401.s001.gz › transit_1.4.0/doc/images/result_table.png]

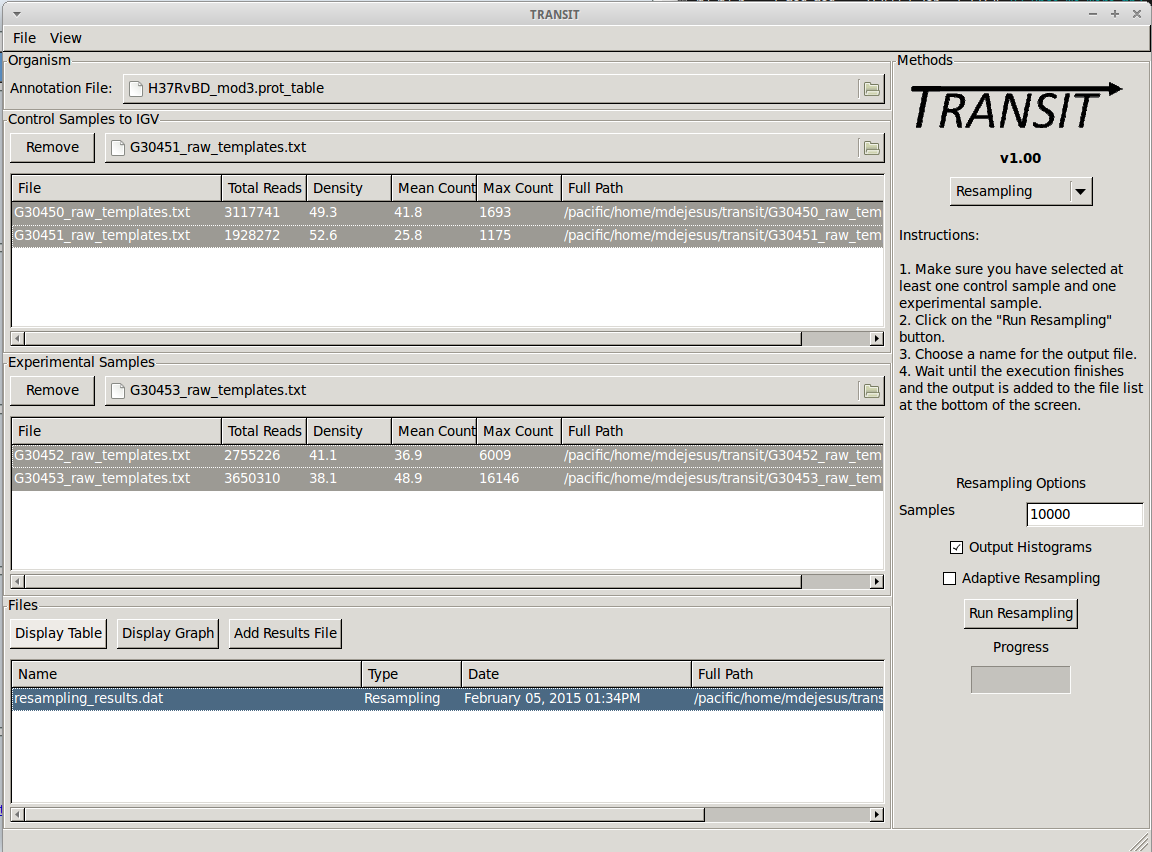

Supplement: S1 Data — Source Code for TRANSIT and TPP, and datasets used to obtain results. Please see the GitHub Repository https://github.com/mad-lab/transit to obtain the latest version of the software. (GZ) [file pcbi.1004401.s001.gz › transit_1.4.0/doc/images/files_list.png]

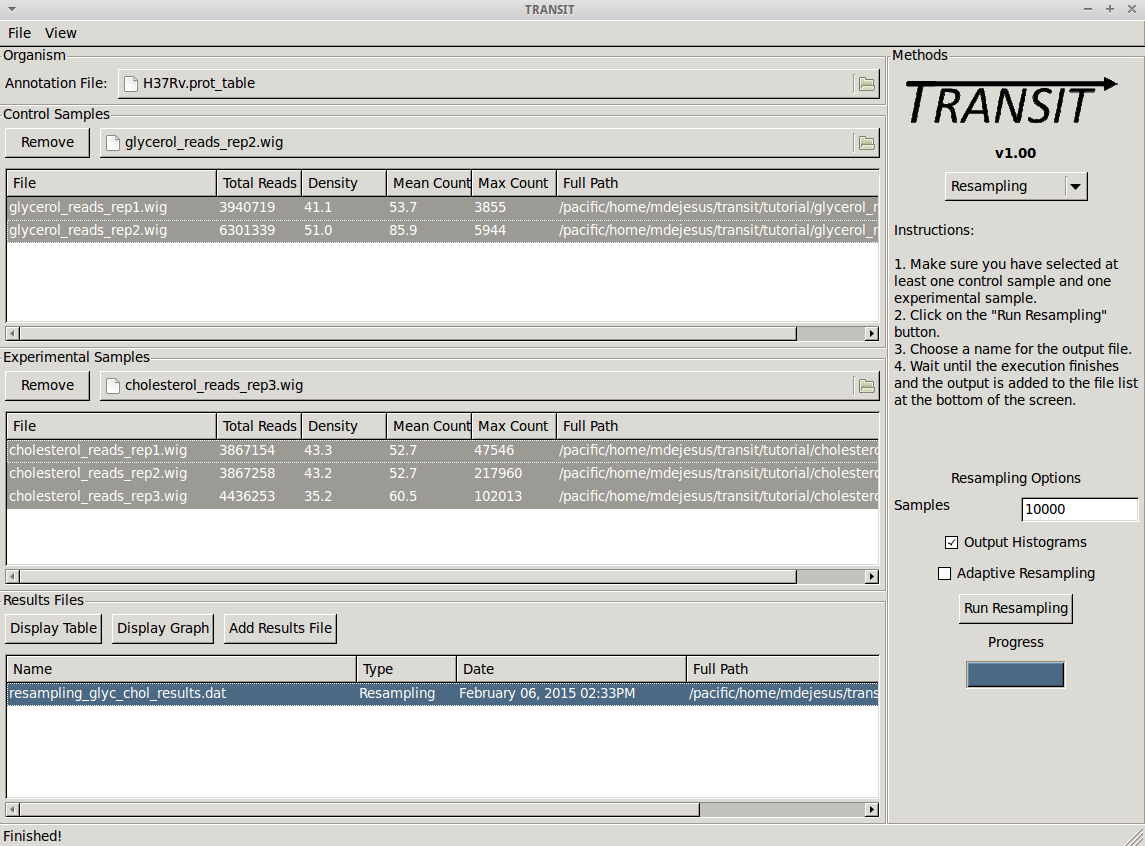

Supplement: S1 Data — Source Code for TRANSIT and TPP, and datasets used to obtain results. Please see the GitHub Repository https://github.com/mad-lab/transit to obtain the latest version of the software. (GZ) [file pcbi.1004401.s001.gz › transit_1.4.0/doc/images/tutorial_resampling_result.png]

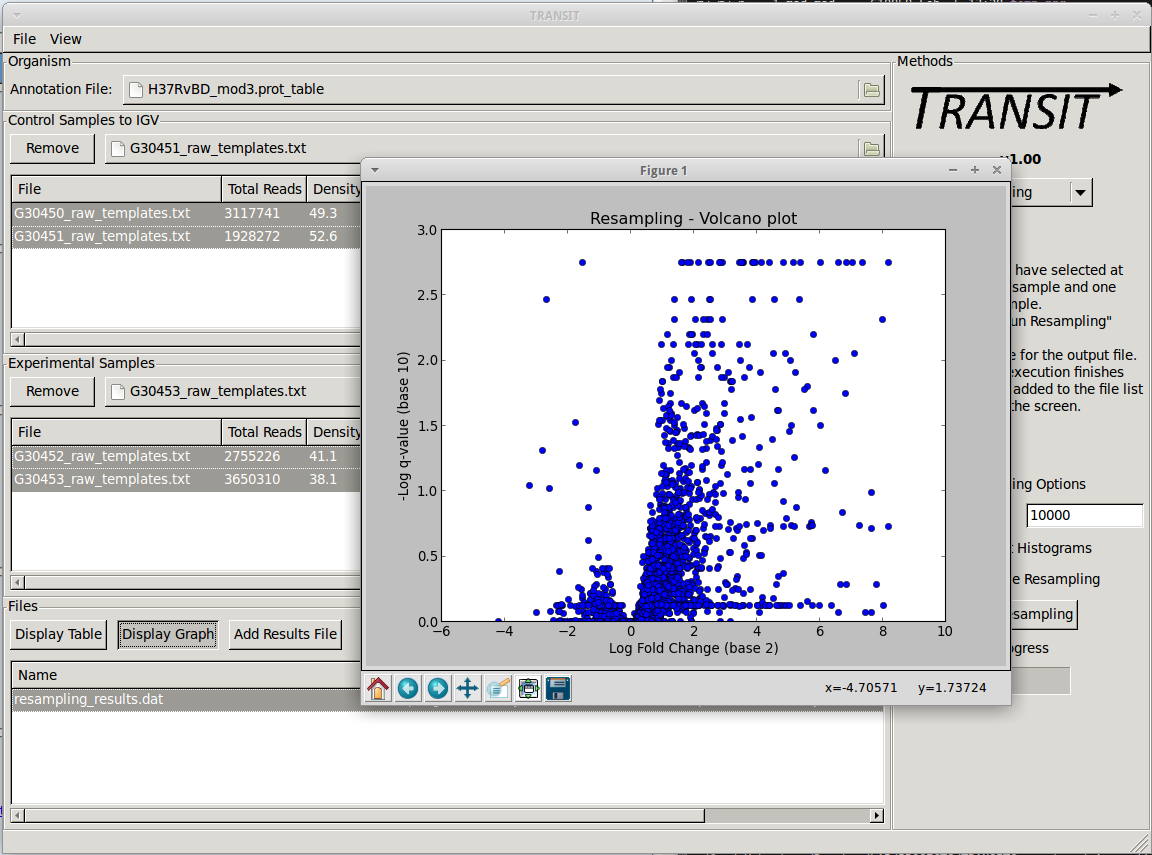

Supplement: S1 Data — Source Code for TRANSIT and TPP, and datasets used to obtain results. Please see the GitHub Repository https://github.com/mad-lab/transit to obtain the latest version of the software. (GZ) [file pcbi.1004401.s001.gz › transit_1.4.0/doc/images/result_volcano_graph.png]

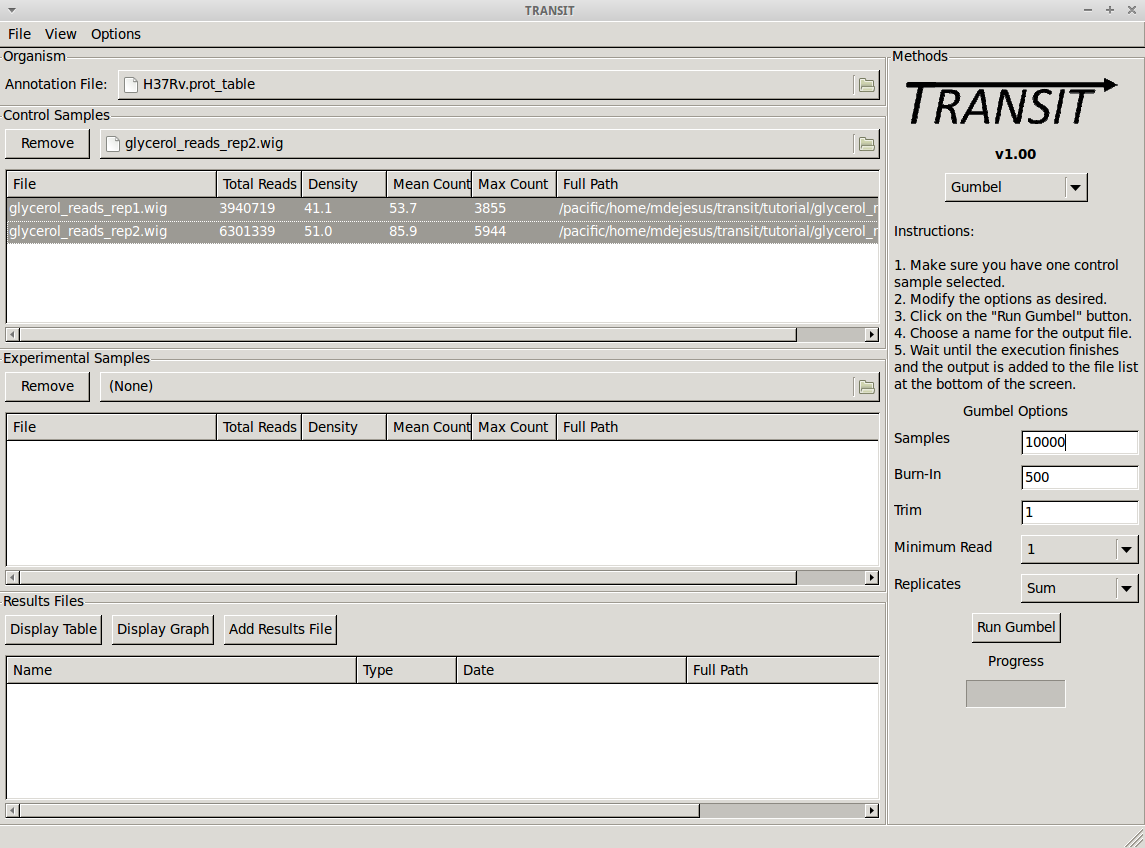

Supplement: S1 Data — Source Code for TRANSIT and TPP, and datasets used to obtain results. Please see the GitHub Repository https://github.com/mad-lab/transit to obtain the latest version of the software. (GZ) [file pcbi.1004401.s001.gz › transit_1.4.0/doc/images/tutorial_gumbel_menu.png]

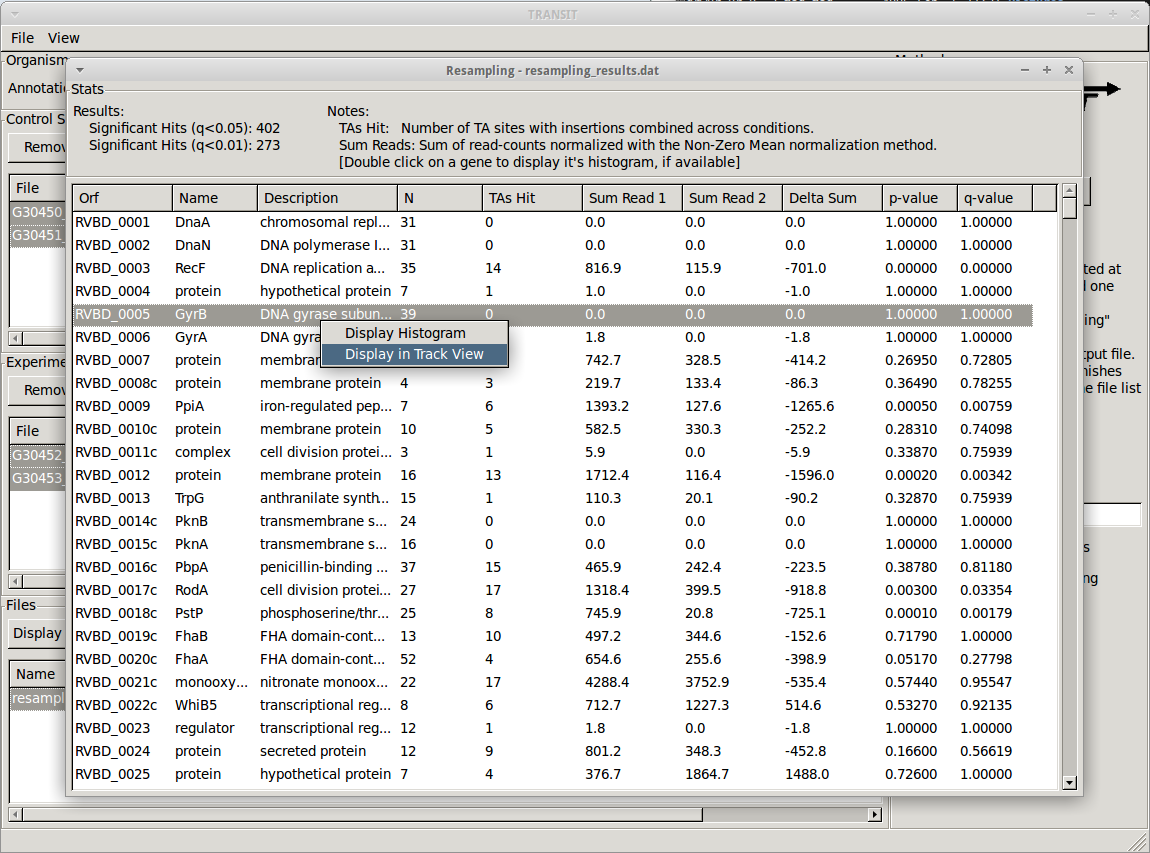

Supplement: S1 Data — Source Code for TRANSIT and TPP, and datasets used to obtain results. Please see the GitHub Repository https://github.com/mad-lab/transit to obtain the latest version of the software. (GZ) [file pcbi.1004401.s001.gz › transit_1.4.0/doc/images/result_table_menu.png]

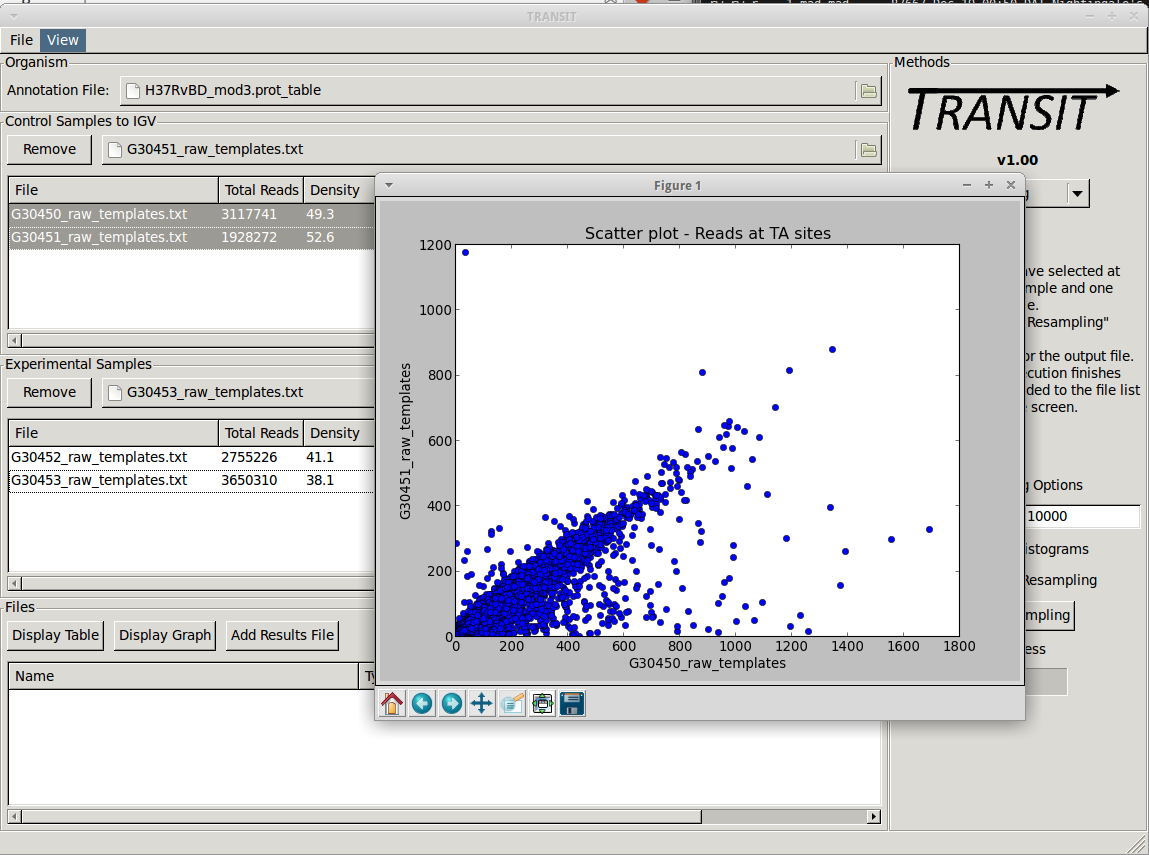

Supplement: S1 Data — Source Code for TRANSIT and TPP, and datasets used to obtain results. Please see the GitHub Repository https://github.com/mad-lab/transit to obtain the latest version of the software. (GZ) [file pcbi.1004401.s001.gz › transit_1.4.0/doc/images/scatter.png]

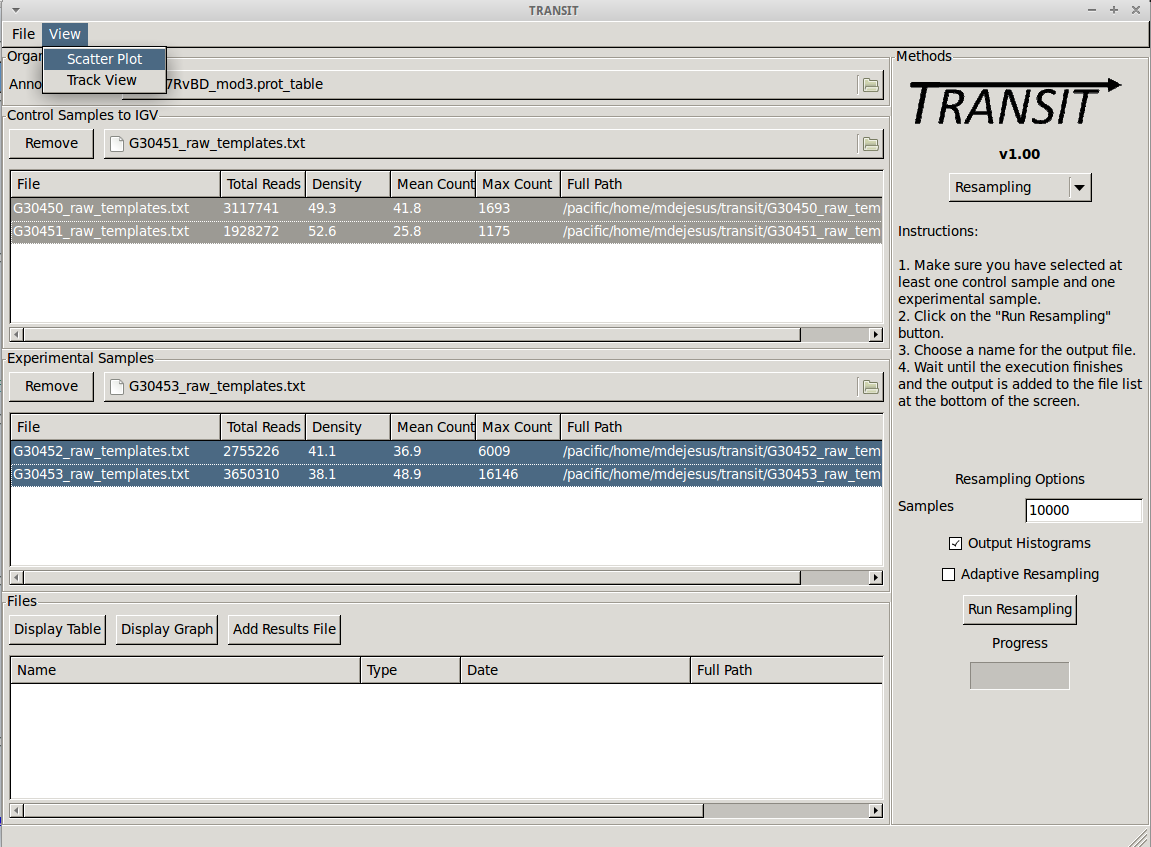

Supplement: S1 Data — Source Code for TRANSIT and TPP, and datasets used to obtain results. Please see the GitHub Repository https://github.com/mad-lab/transit to obtain the latest version of the software. (GZ) [file pcbi.1004401.s001.gz › transit_1.4.0/doc/images/view_menu.png]

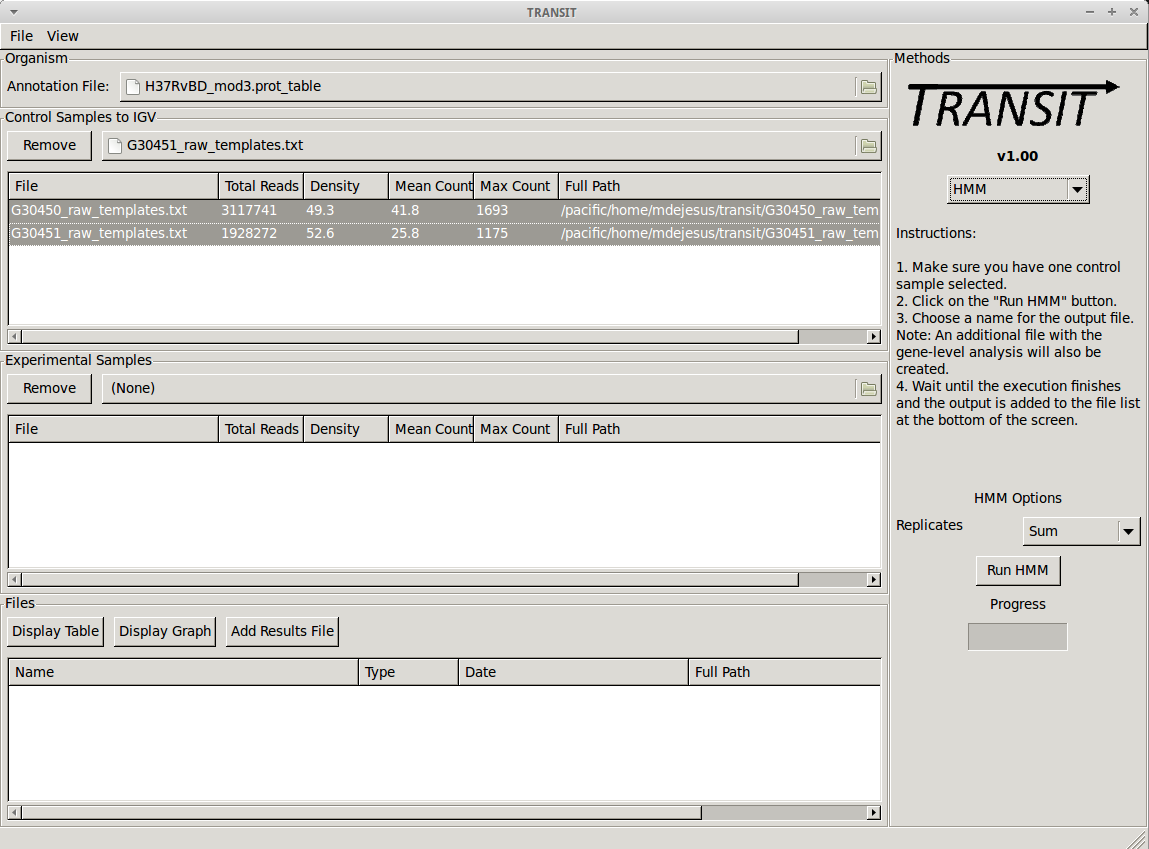

Supplement: S1 Data — Source Code for TRANSIT and TPP, and datasets used to obtain results. Please see the GitHub Repository https://github.com/mad-lab/transit to obtain the latest version of the software. (GZ) [file pcbi.1004401.s001.gz › transit_1.4.0/doc/images/hmm_g0g1.png]

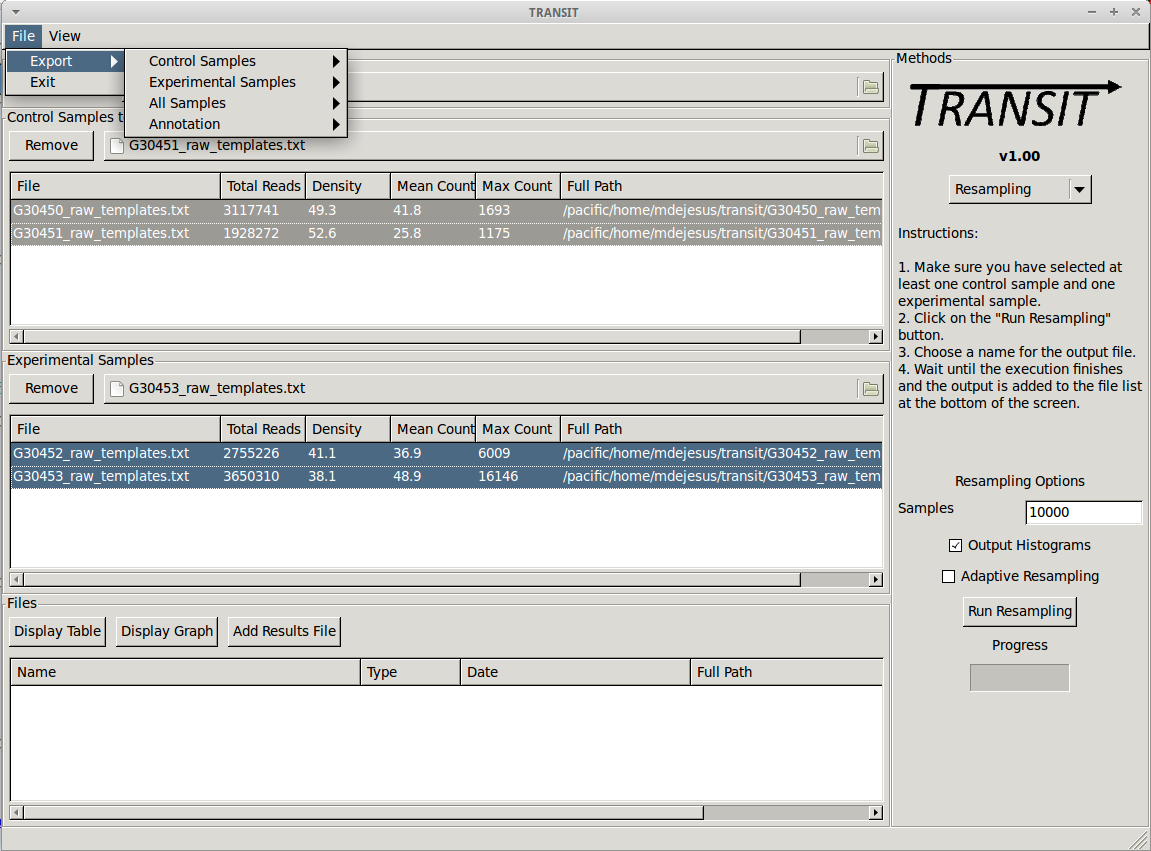

Supplement: S1 Data — Source Code for TRANSIT and TPP, and datasets used to obtain results. Please see the GitHub Repository https://github.com/mad-lab/transit to obtain the latest version of the software. (GZ) [file pcbi.1004401.s001.gz › transit_1.4.0/doc/images/export_menu.png]

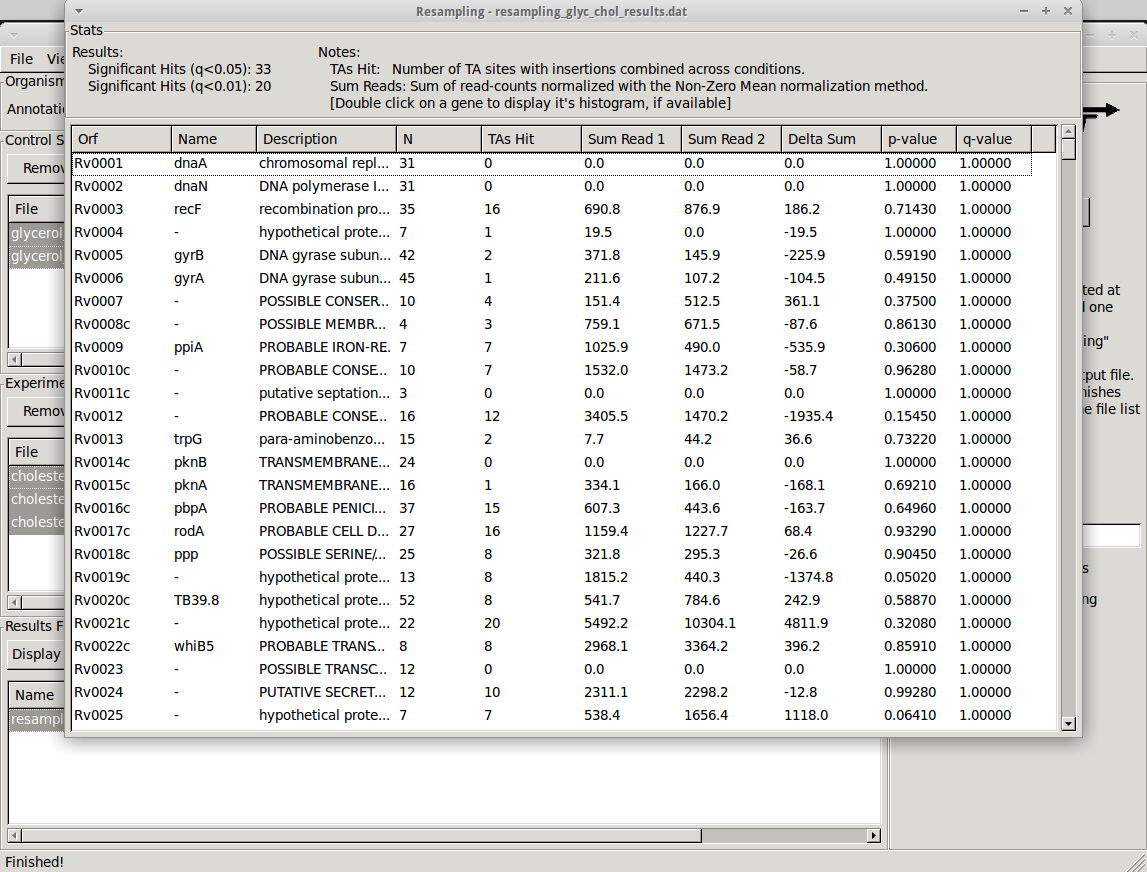

Supplement: S1 Data — Source Code for TRANSIT and TPP, and datasets used to obtain results. Please see the GitHub Repository https://github.com/mad-lab/transit to obtain the latest version of the software. (GZ) [file pcbi.1004401.s001.gz › transit_1.4.0/doc/images/tutorial_resampling_file.png]

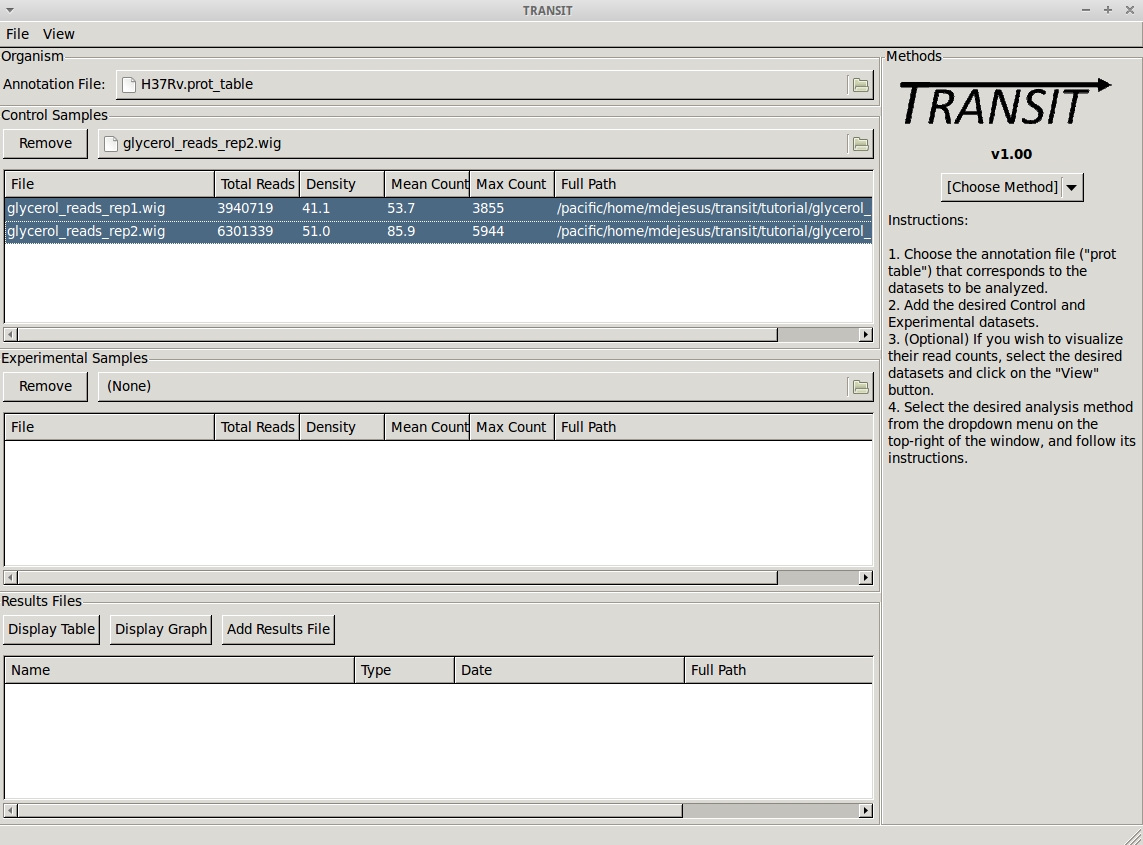

Supplement: S1 Data — Source Code for TRANSIT and TPP, and datasets used to obtain results. Please see the GitHub Repository https://github.com/mad-lab/transit to obtain the latest version of the software. (GZ) [file pcbi.1004401.s001.gz › transit_1.4.0/doc/images/tutorial_glyc_ctrl.png]

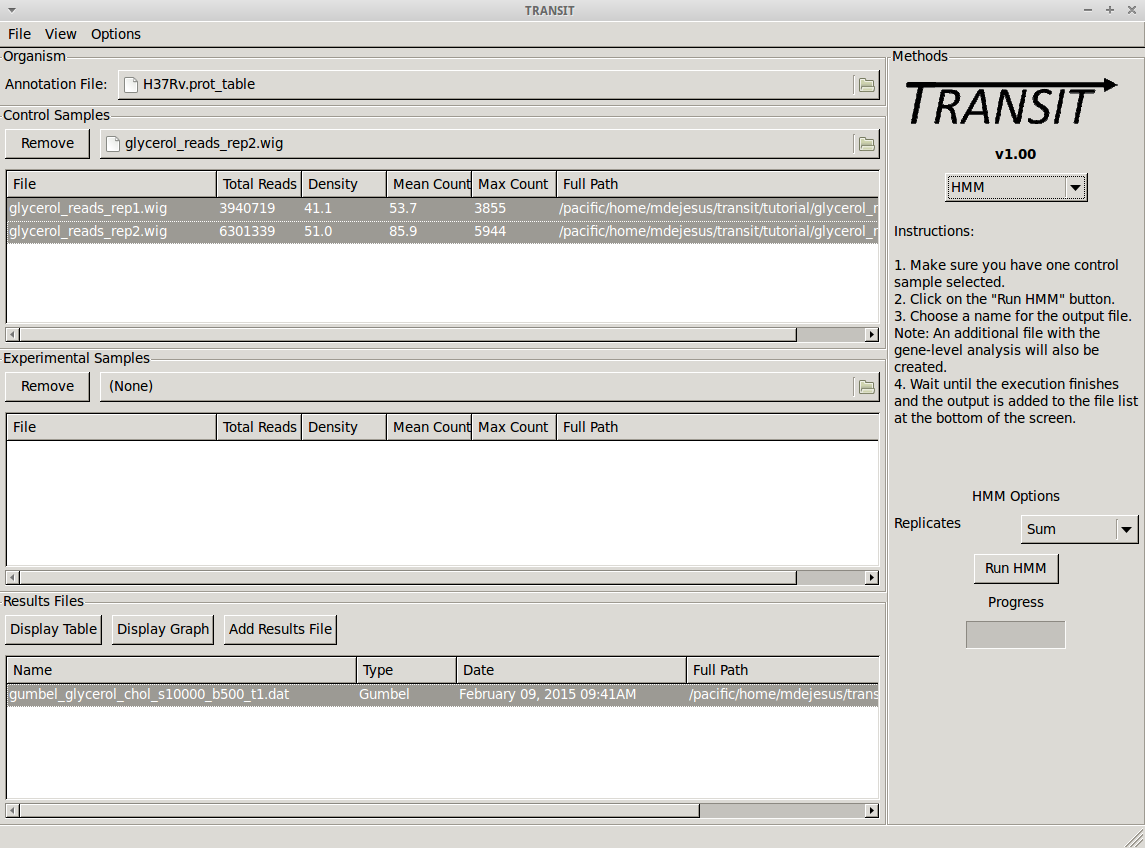

Supplement: S1 Data — Source Code for TRANSIT and TPP, and datasets used to obtain results. Please see the GitHub Repository https://github.com/mad-lab/transit to obtain the latest version of the software. (GZ) [file pcbi.1004401.s001.gz › transit_1.4.0/doc/images/tutorial_hmm_menu.png]

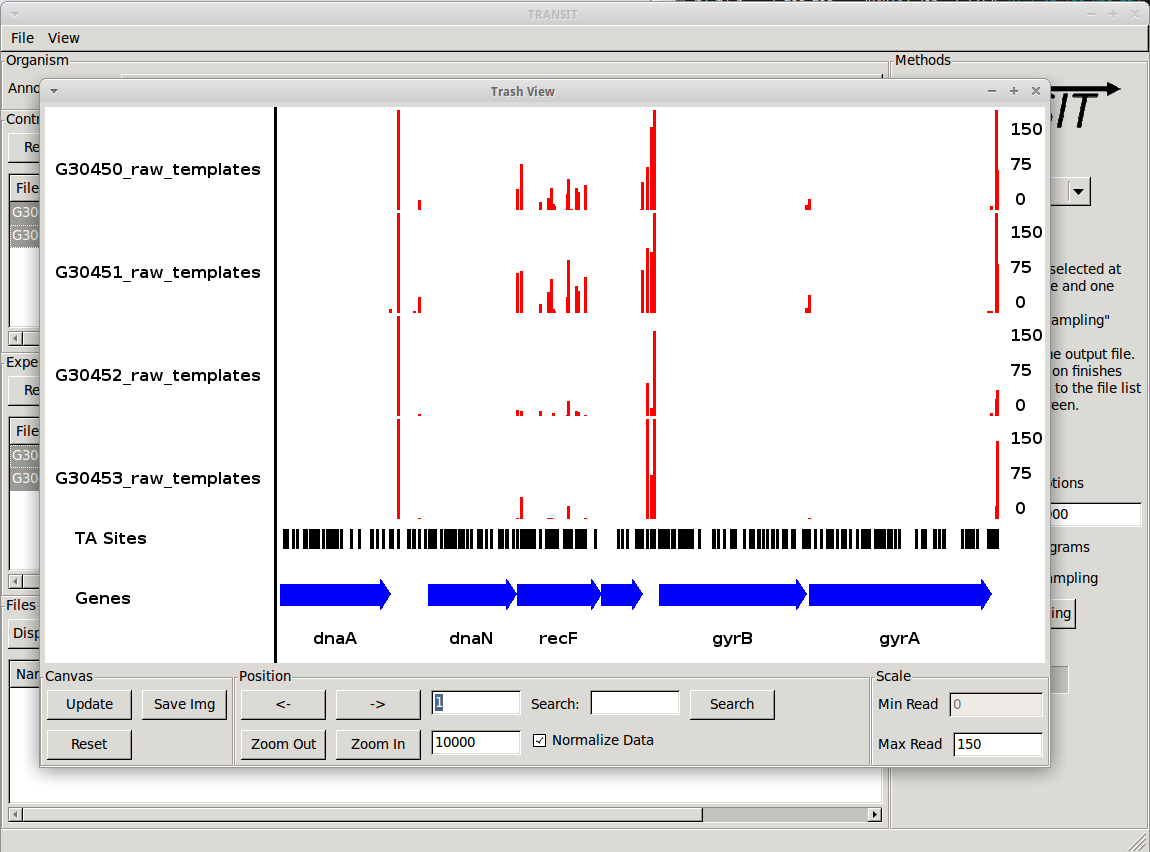

Supplement: S1 Data — Source Code for TRANSIT and TPP, and datasets used to obtain results. Please see the GitHub Repository https://github.com/mad-lab/transit to obtain the latest version of the software. (GZ) [file pcbi.1004401.s001.gz › transit_1.4.0/doc/images/track_view.png]

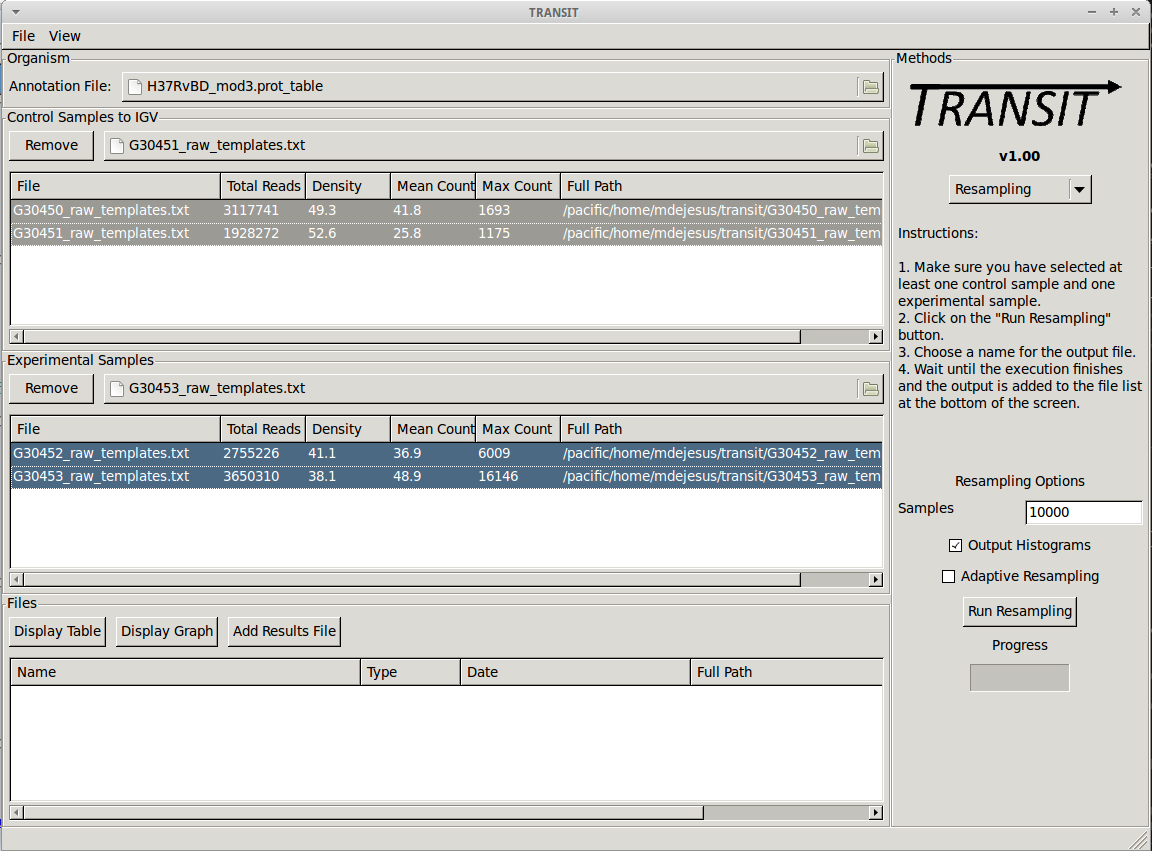

Supplement: S1 Data — Source Code for TRANSIT and TPP, and datasets used to obtain results. Please see the GitHub Repository https://github.com/mad-lab/transit to obtain the latest version of the software. (GZ) [file pcbi.1004401.s001.gz › transit_1.4.0/doc/images/resampling_g0g1_g2g3.png]

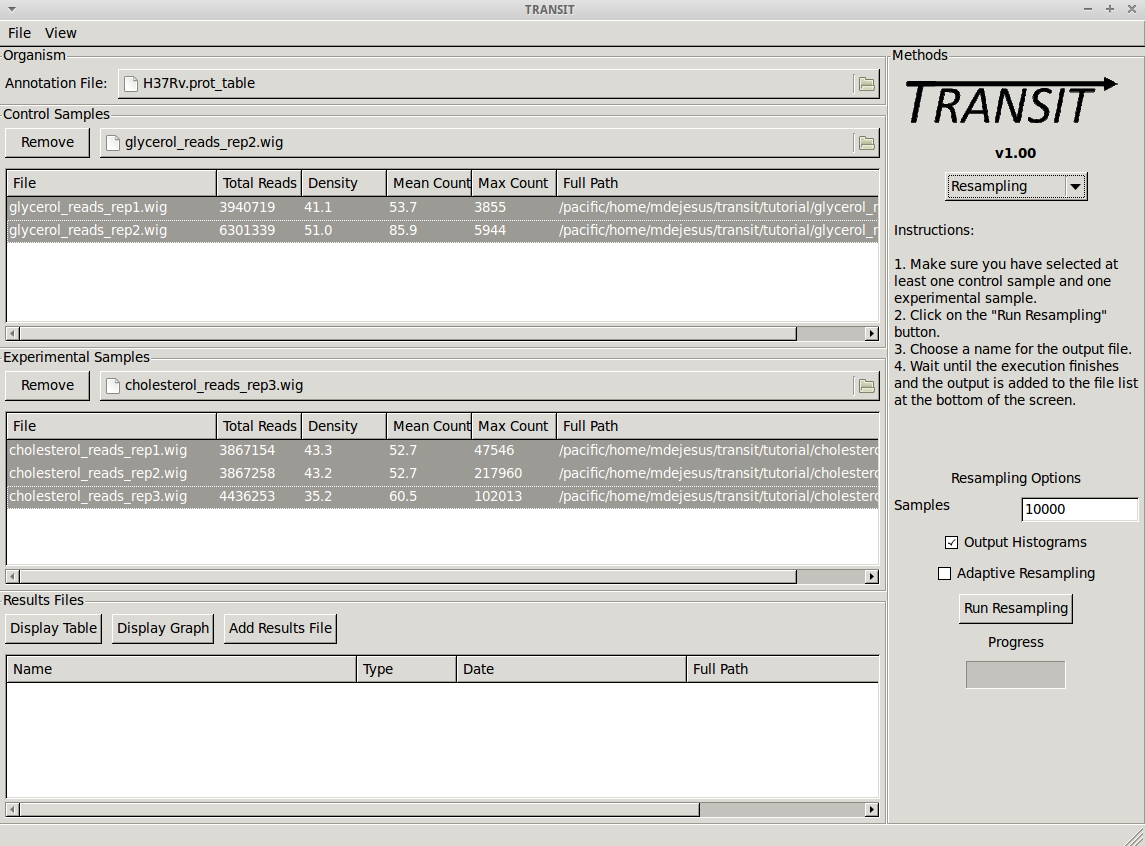

Supplement: S1 Data — Source Code for TRANSIT and TPP, and datasets used to obtain results. Please see the GitHub Repository https://github.com/mad-lab/transit to obtain the latest version of the software. (GZ) [file pcbi.1004401.s001.gz › transit_1.4.0/doc/images/tutorial_resampling.png]

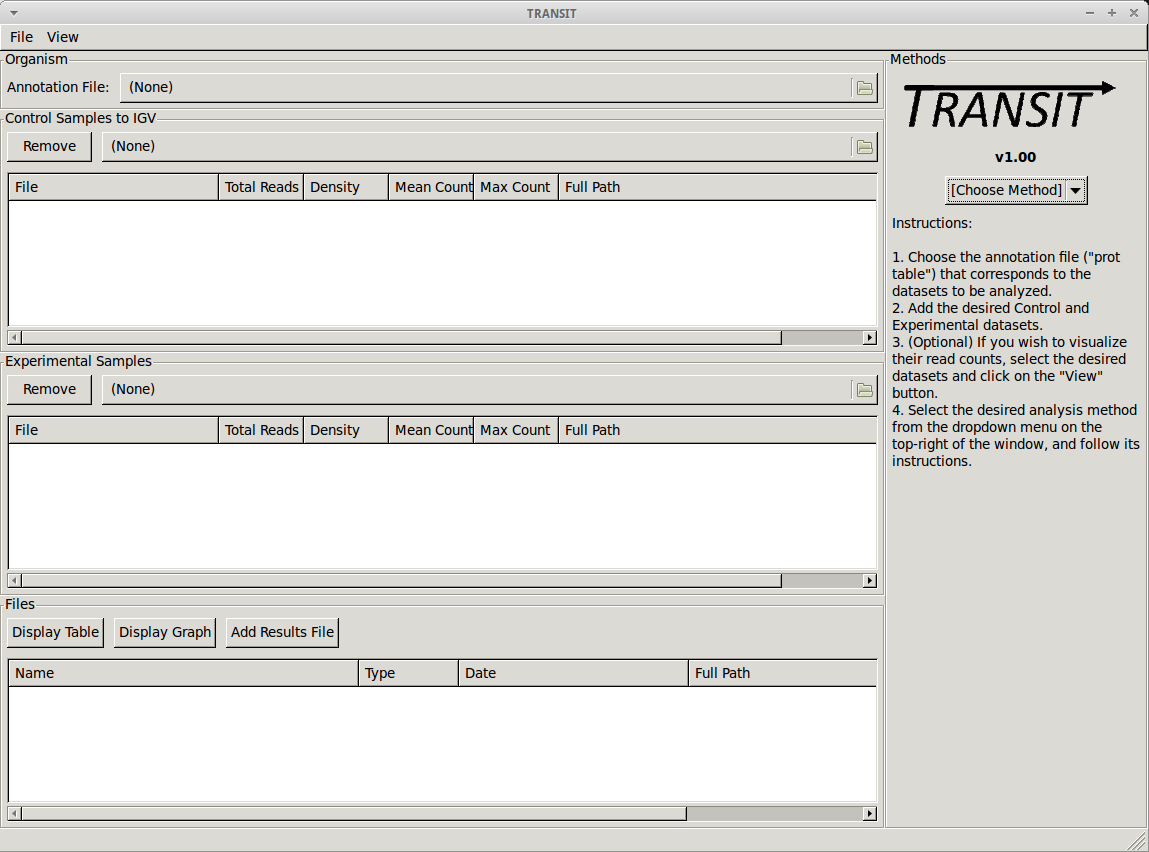

Supplement: S1 Data — Source Code for TRANSIT and TPP, and datasets used to obtain results. Please see the GitHub Repository https://github.com/mad-lab/transit to obtain the latest version of the software. (GZ) [file pcbi.1004401.s001.gz › transit_1.4.0/doc/images/main.png]

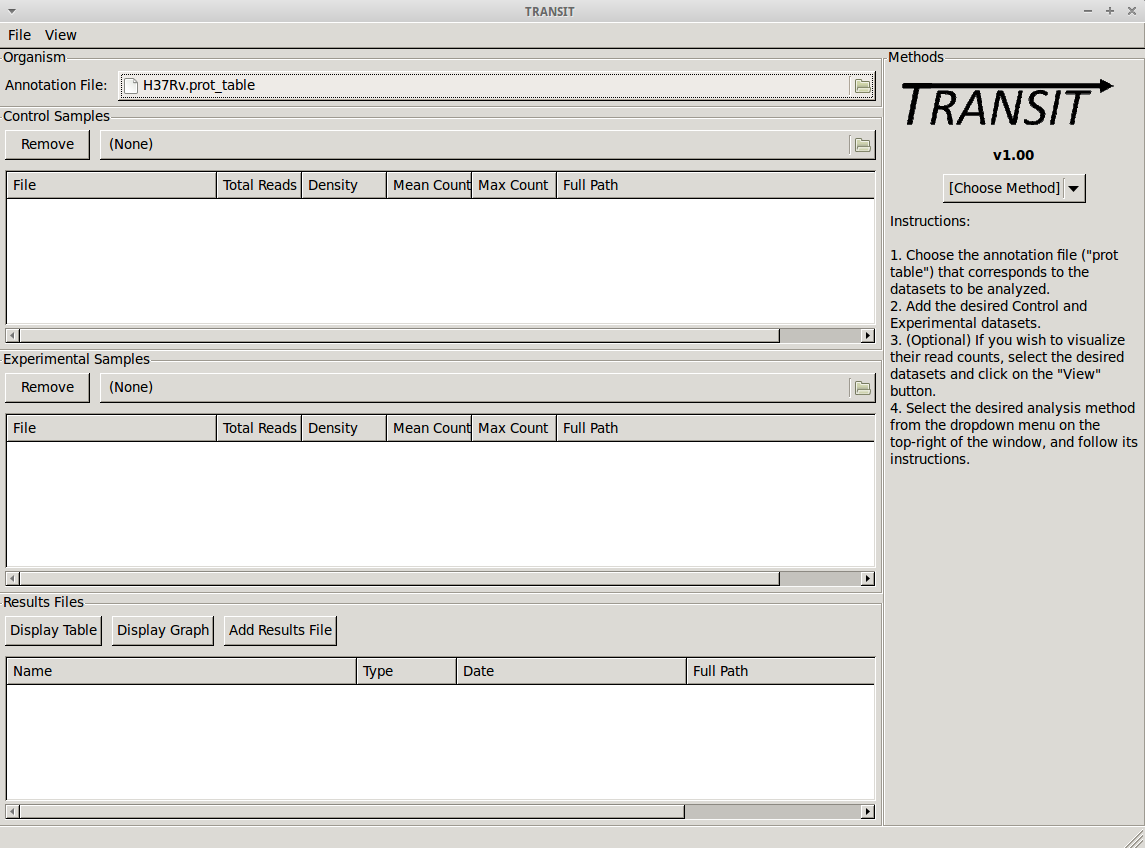

Supplement: S1 Data — Source Code for TRANSIT and TPP, and datasets used to obtain results. Please see the GitHub Repository https://github.com/mad-lab/transit to obtain the latest version of the software. (GZ) [file pcbi.1004401.s001.gz › transit_1.4.0/doc/images/tutorial_annotation.png]

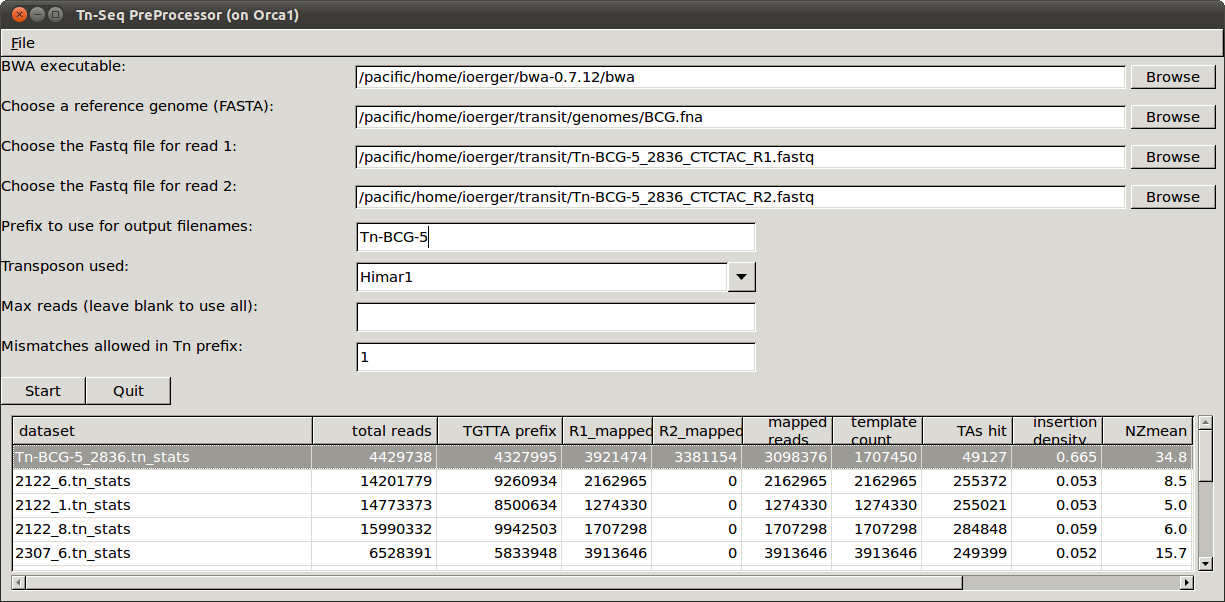

Supplement: S1 Data — Source Code for TRANSIT and TPP, and datasets used to obtain results. Please see the GitHub Repository https://github.com/mad-lab/transit to obtain the latest version of the software. (GZ) [file pcbi.1004401.s001.gz › transit_1.4.0/doc/images/TPP-screenshot.png]

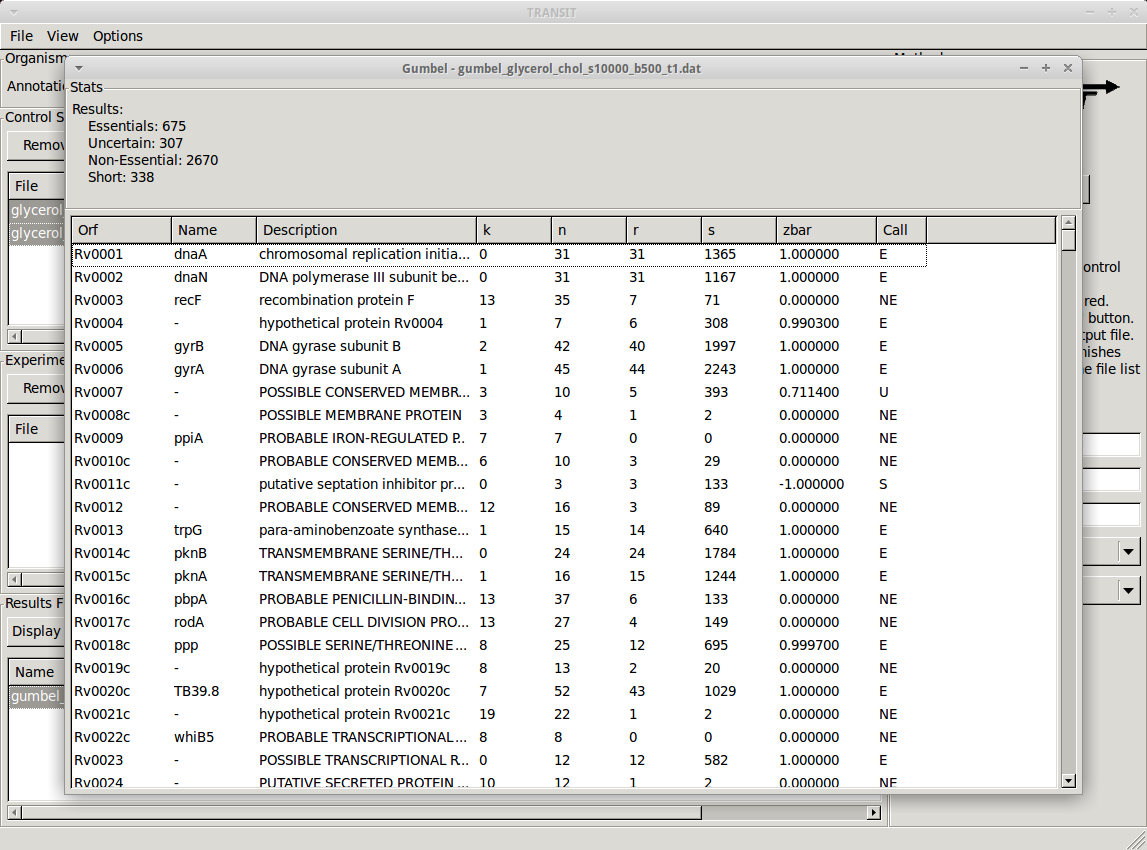

Supplement: S1 Data — Source Code for TRANSIT and TPP, and datasets used to obtain results. Please see the GitHub Repository https://github.com/mad-lab/transit to obtain the latest version of the software. (GZ) [file pcbi.1004401.s001.gz › transit_1.4.0/doc/images/tutorial_gumbel_file.png]

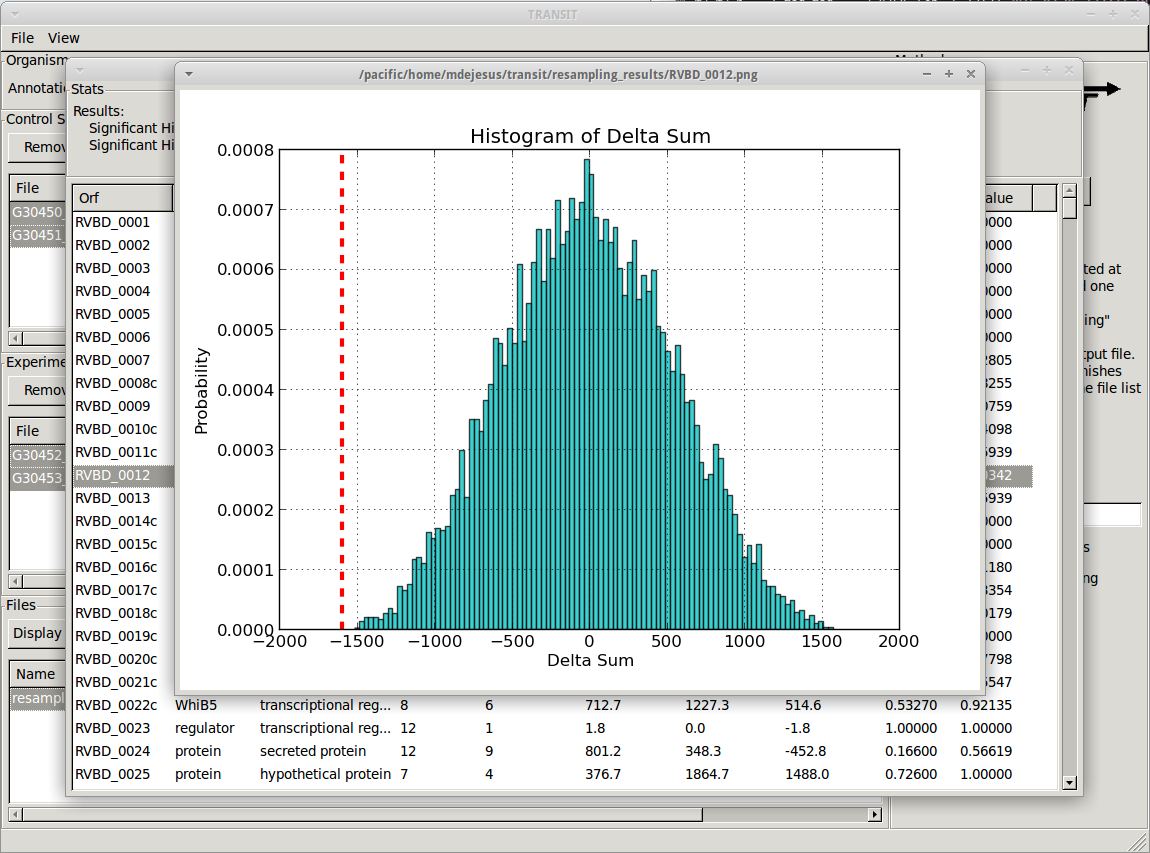

Supplement: S1 Data — Source Code for TRANSIT and TPP, and datasets used to obtain results. Please see the GitHub Repository https://github.com/mad-lab/transit to obtain the latest version of the software. (GZ) [file pcbi.1004401.s001.gz › transit_1.4.0/doc/images/result_table_histogram.png]

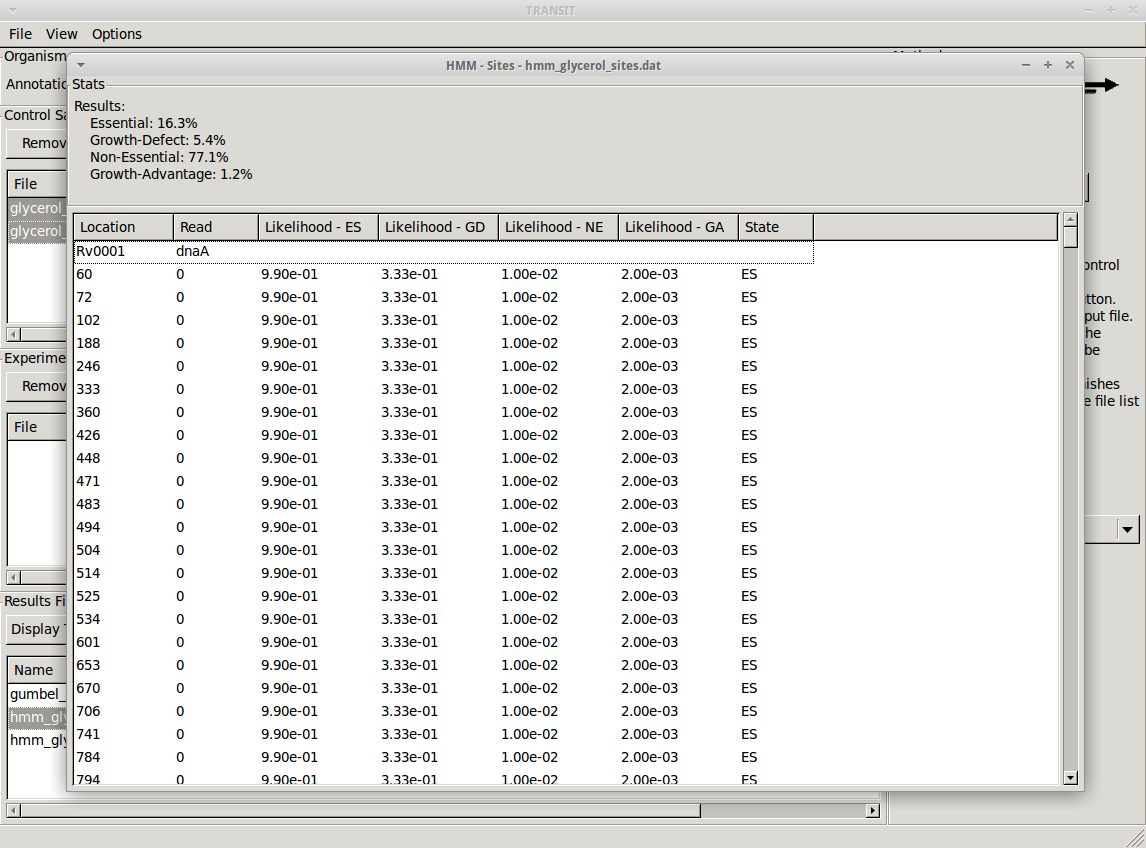

Supplement: S1 Data — Source Code for TRANSIT and TPP, and datasets used to obtain results. Please see the GitHub Repository https://github.com/mad-lab/transit to obtain the latest version of the software. (GZ) [file pcbi.1004401.s001.gz › transit_1.4.0/doc/images/tutorial_hmm_sites.png]

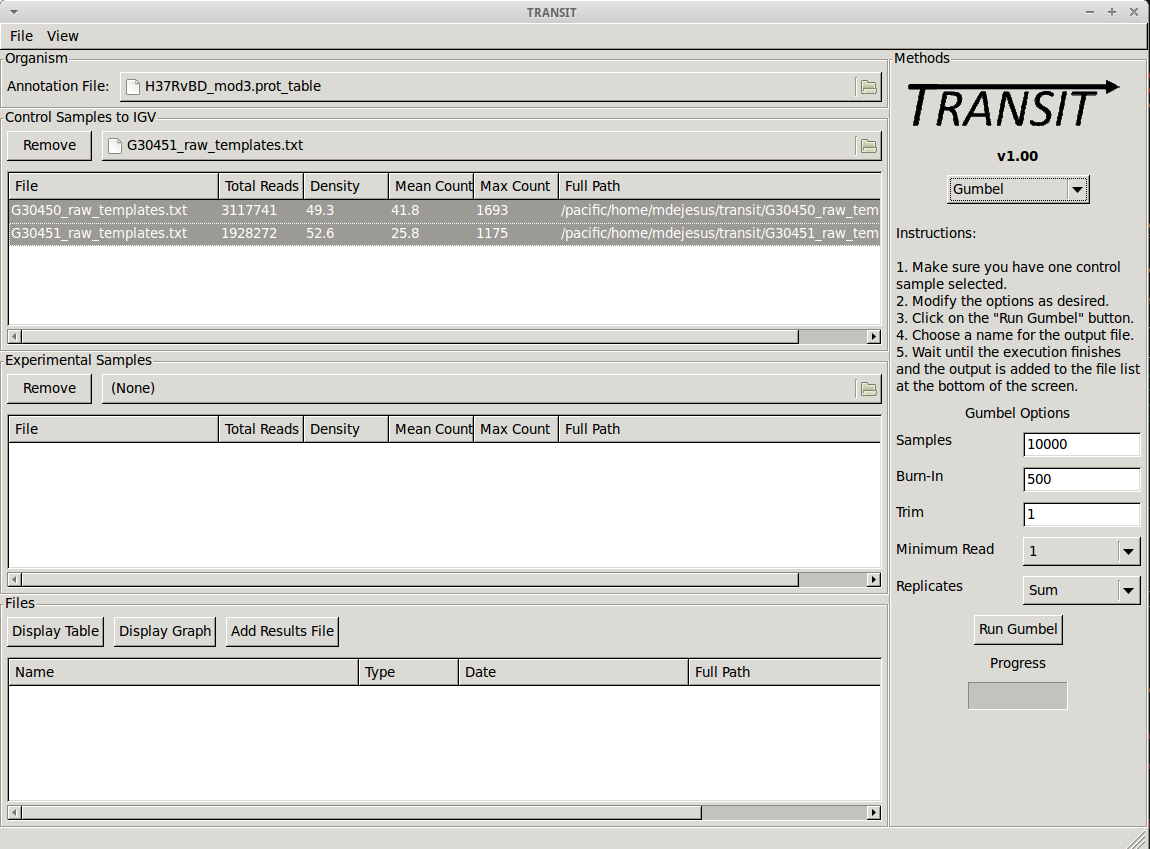

Supplement: S1 Data — Source Code for TRANSIT and TPP, and datasets used to obtain results. Please see the GitHub Repository https://github.com/mad-lab/transit to obtain the latest version of the software. (GZ) [file pcbi.1004401.s001.gz › transit_1.4.0/doc/images/gumbel_g0g1.png]

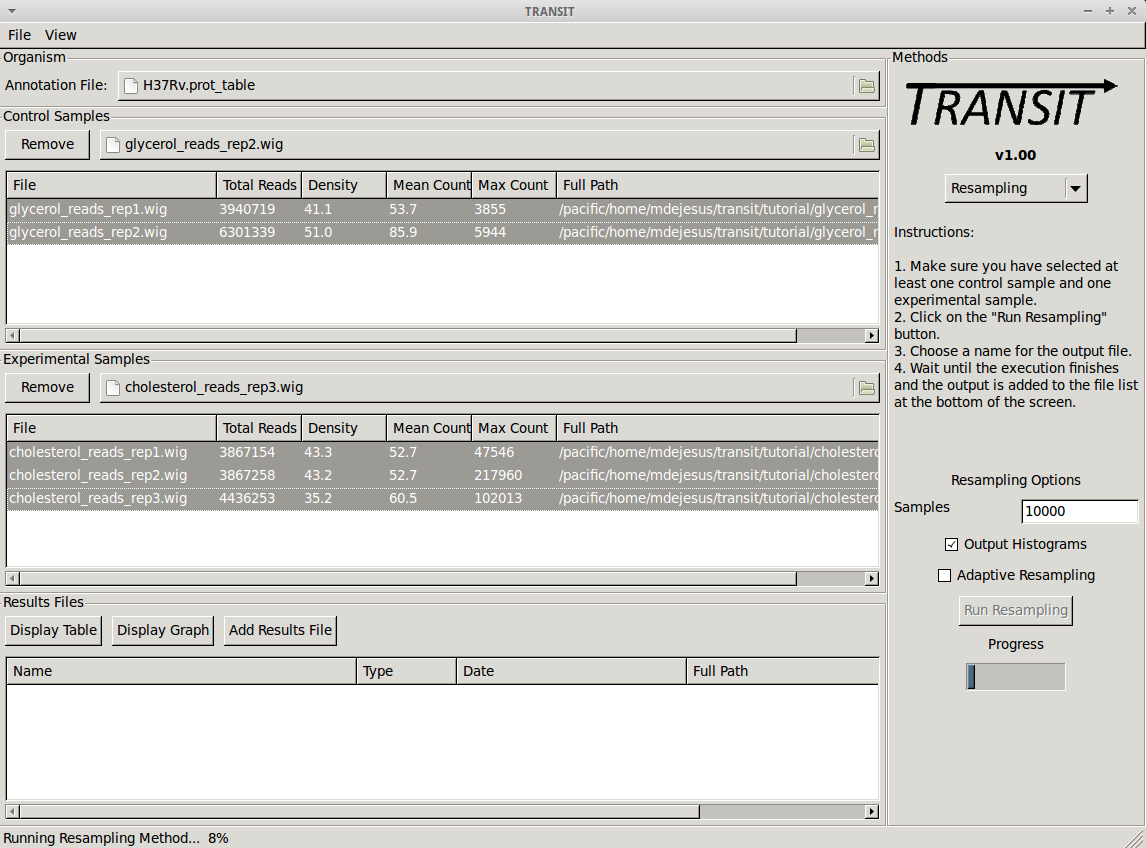

Supplement: S1 Data — Source Code for TRANSIT and TPP, and datasets used to obtain results. Please see the GitHub Repository https://github.com/mad-lab/transit to obtain the latest version of the software. (GZ) [file pcbi.1004401.s001.gz › transit_1.4.0/doc/images/tutorial_resampling_running.png]

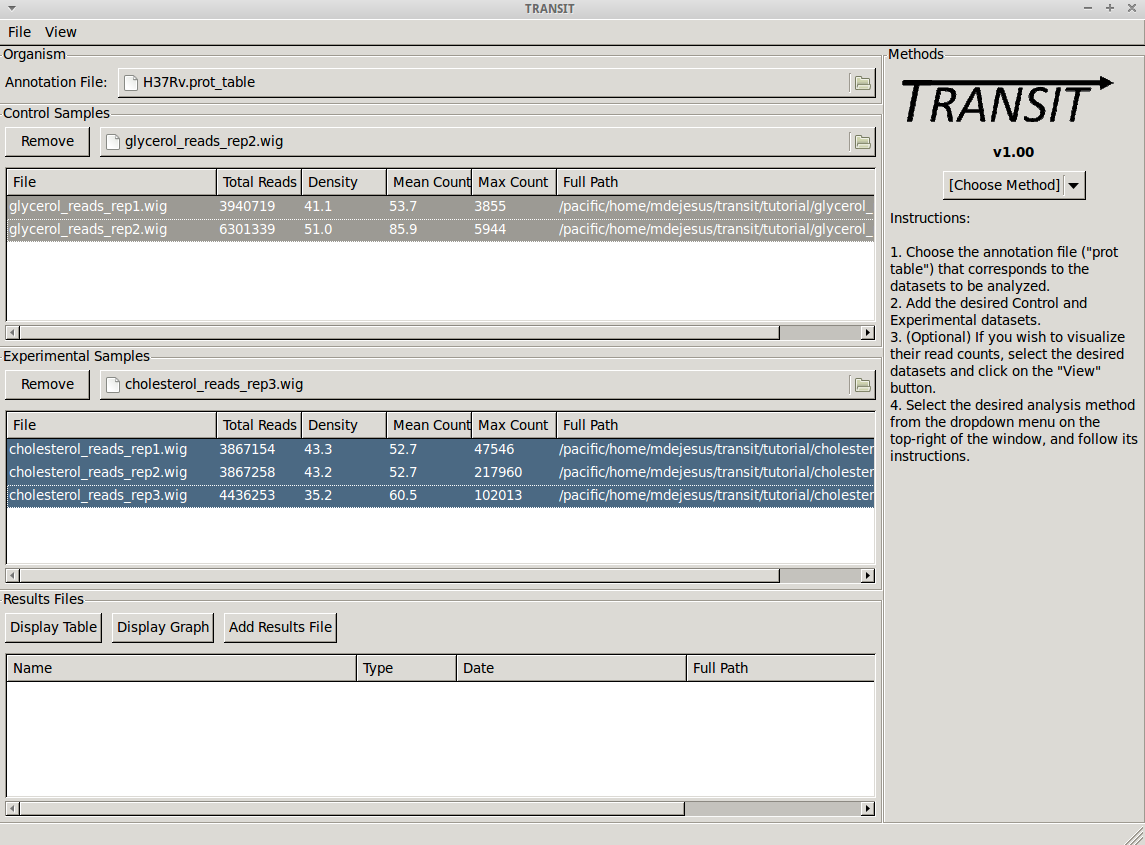

Supplement: S1 Data — Source Code for TRANSIT and TPP, and datasets used to obtain results. Please see the GitHub Repository https://github.com/mad-lab/transit to obtain the latest version of the software. (GZ) [file pcbi.1004401.s001.gz › transit_1.4.0/doc/images/tutorial_chol_exp.png]
